# eNose technologies in the detection of cancer: a systematic review and meta-analysis

**DOI:** 10.1093/oncolo/oyag016

**Published:** 2026-01-28

**Authors:** Eitan J Neugut, Alfred I Neugut

**Affiliations:** Department of Statistics, Columbia University, New York, NY 10027, United States; Department of Medicine and Herbert Irving Comprehensive Cancer Center, Vagelos College of Physicians & Surgeons, Columbia University, New York, NY 10032, United States; Department of Epidemiology, Mailman School of Public Health, Columbia University, New York, NY 10032, United States

**Keywords:** eNose, electronic nose, cancer detection, sensor array, volatile organic compounds, meta-analysis

## Abstract

**Background:**

In 2004, researchers in the United Kingdom were able to train six dogs to distinguish between urine samples from bladder cancer patients and healthy controls using smell, achieving a detection rate of 41% (95% confidence interval [CI]: 23-58%), far above the 14% expected by chance. After numerous subsequent studies validated that the odor of breath and urine samples could be used by dogs to detect cancer, researchers pivoted to electronic noses (eNoses), sensor-based systems that mimic the sense of smell using arrays of chemical detectors. In this study, we review the potential efficacy of eNoses in the detection of selected cancer types in human biological samples.

**Methods:**

We identified and performed a meta-analysis on 37 studies of eNose technology, comprising 1 365 cancer patients and 2 249 control subjects. We calculated the pooled sensitivity and specificity stratified by cancer type, sample type, and sensor type. Meta-regressions were conducted on these variables as well as the number of sensors used in the sensor array.

**Results:**

All six cancer types analyzed—breast, colorectal, gastric, lung, ovarian, and prostate—achieved pooled sensitivities and specificities above 70%, with most around 85%. The overall pooled sensitivity was 85.9% (95% CI: 82.3-88.9%) and specificity was 83.6% (95% CI: 78.6-87.7%). Meta-regression revealed that the number of sensors in the sensor arrays, up to 15 sensors, was predictive of sensitivity with *P*_FDR_ < 0.001.

**Conclusion:**

This analysis found that eNoses constitute a promising tool in the early detection of cancer. However, more research is necessary before it can be introduced into clinical settings.

Implications for Practice Electronic noses (eNoses)—devices that mimic the sense of smell to analyze breath, urine, or other samples—show about 85% sensitivity and specificity in the early detection of a wide range of cancers. A single inexpensive, portable eNose could potentially transform early detection by accurately identifying multiple cancer types. However, more research is necessary before it can be introduced into clinical settings.

## Introduction

In this review, we examine the efficacy and potential of electronic nose (eNose) technology in the detection of various types of cancer. In addition, we assess the performance of different sensor types included in the eNose, the number of sensors, and the sample type analyzed.

### Origin in canine olfactory studies

The use of olfactory detection for cancer dates back to a 1989 case report when a dog’s unusual interest in a spot on its owner’s hand led to the detection of a melanoma at King’s College Hospital in London.^1^ Interest grew, and in 2004, researchers in the United Kingdom trained six dogs to distinguish between urine samples from bladder cancer patients and healthy controls, achieving a detection rate of 41% (95% CI: 23-58%), far above the 14% expected by chance.^2^

Canine experiments were subsequently conducted for various cancer types, including bladder,^2^^,^^3^ breast,^4–6^ colorectal,^7^ hepatic,^8^ lung,^4^^,^^5^^,^^9–18^ melanoma,^5^ osteosarcoma,^19^ ovarian,^20^ and prostate,^21–24^ with many reporting sensitivities and specificities >90%. For example, a notable 2015 study by Taverna et al.^25^ at the Humanitas Clinical and Research Center in Milan, Italy trained two German Shepherds to detect prostate cancer and reported impressive results: 99.3% sensitivity and 98.2% specificity on urine samples from 162 cancer patients and 310 controls.

While the use of canines faced significant practical barriers, including concerns about the FDA’s willingness to authorize animal-based diagnostic methods, logistical challenges in costs and scaling, and ethical concerns,^26^^,^^27^ the dog studies revealed that the scent profile of breath, urine, and other samples contain chemical patterns that indicate the cancer status of an individual, leading researchers to new approaches for cancer detection.

### Sensor arrays for cancer detection

eNoses are biomimetic systems designed to replicate the sense of smell with the use of an array of chemical sensors. Originally developed for applications in the food and beverage industries to assess product quality and aroma profiles, these technologies have expanded into fields, such as environmental monitoring and medical diagnostics.^28^^,^^29^ In particular, their ability to detect and analyze complex chemical patterns in biological samples has made them promising tools for non-invasive cancer detection.^30^

**Figure 1.**
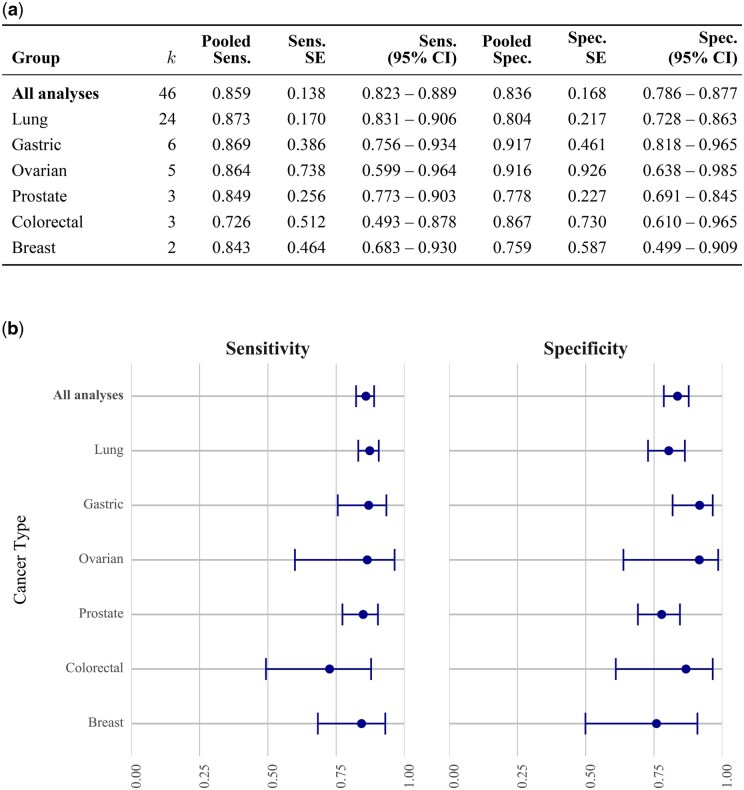
Cancer type stratified meta-analysis. (a) Pooled sensitivity and specificity with 95% confidence intervals by cancer type. Some studies performed more than one classification analysis; *k* denotes the number of analyses in each subcategory. (b) Box-and-whisker plots showing pooled estimates with 95% confidence intervals.

eNoses function by generating a collective response to a sample’s chemical composition, forming a “signature” that can be analyzed to detect specific patterns related to disease.^31^ Sensor arrays use a combination of chemical sensors to detect volatile organic compounds (VOCs) in biological samples, such as breath, urine, blood, and saliva. Each sensor in the array responds to a different set of compounds based on their chemical properties, producing a collective “fingerprint” that can be analyzed with the use of statistical or machine learning algorithms. The sensor data is then used in classification algorithms to detect patterns of VOCs that correlate with various cancers due to metabolic changes associated with the particular cancer (Figure 1).^30^^,^^32^^,^^33^

### Comparison to chemical analyzers

Many studies have used traditional chemical analyzers, such as gas chromatography-mass spectrometry (GC-MS), proton transfer reaction-mass spectrometry (PTR-MS), field asymmetric ion mobility spectrometry (FAIMS), etc, for the detection and analysis of VOCs in biological samples.^34–36^ These methods involve a two-step process: first, the separation of compounds in the sample, and second, the identification of each compound based on its chemical properties.^37^

In contrast, rather than isolating and identifying individual compounds, eNoses respond to VOCs collectively, providing more of a general sense of the “smell” profile of the sample.^38^ This approach, although less granular, offers advantages in speed, simplicity, affordability, and portability of the device, making them better suited for use in clinical settings.

While studies utilizing chemical analyzers have been able to identify individual compounds that correlate with cancer, there is a lack of consensus about the specific compounds that should be used for cancer diagnosis. Gouzerh et al.^33^ reviewed 118 studies and found that 458 unique compounds were identified, but only 116 were found in more than one study.

Moreover, studies have reported conflicting associations for the same compounds.^39^ For example, Gouzerh et al.^33^ found that, of the five most commonly reported compounds correlated with cancers (hexanal, toluene, styrene, ethylbenzene, acetone), 30 studies reported increased concentrations in cancer patients, while 11 studies showed decreased levels.

These inconsistencies reflect the complexity of cancer-related metabolic changes, which involve broader patterns rather than individual compounds. Thus, eNose technologies, which capture these complex chemical patterns, may offer a more effective approach for cancer screening.

### Sensor array technologies

While all studies in this review used eNose systems, the sensor arrays varied substantially in both the numbers and types of sensors. These sensor types differ in detection mechanism and detection limit, range of measurement, stability, and cost. A summary of common sensor types is in Table 1.^40–46^

**Table 1.** Overview of sensor types used in sensor arrays.

Operating principle	Sensor type	Sensor subtypes	Analyte phase	Detection limit	Description
**Chemical reaction-based**	Chemiresistive sensors	Metal-oxide semiconductor (MOS)	Gas	∼1 ppm	Detect gases by changing electrical resistance when gas interacts with the sensing material.
		Polymer-based	Gas	∼0.1 ppm	
		Nanomaterial-based	Gas	∼0.1 ppm	
	Electrochemical sensors	Voltammetric	Liquid	∼1 ppm	Measure changes in current generated by applying varying voltages to electrodes in contact with the sample.
		Amperometric	Liquid	∼1 ppm	Measure current produced by redox reactions at the working electrode.
		Potentiometric	Liquid	∼1 ppm	Measure changes in electrical potential between a working and reference electrode.
		Conductometric	Liquid	∼100 ppm	Measure changes in conductivity due to ionic composition changes.
		Impedimetric	Liquid	∼10 ppm	Measure impedance (resistance and reactance) at the sensor surface.
	Catalytic combustion sensors		Gas	∼1000 ppm	Detect combustible gases through a catalytic oxidation reaction that increases the sensor’s temperature, altering the sensor’s resistance.
**Mass-based**	Quartz crystal microbalance (QCM) sensors		Gas/liquid	∼0.05 ppm	Measure mass changes on a quartz crystal due to gas adsorption.
	Surface acoustic wave (SAW) sensors		Gas	∼0.01 ppm	Detect gases by measuring changes in the velocity of surface acoustic waves due to gas adsorption.
**Optical**	Photoionization detectors (PID)		Gas	∼10 ppm	Use UV light to ionize gas molecules and measure ionized gas concentrations.
	Fluorometric sensors		Gas/liquid	∼0.025 ppm	Detect gases based on fluorescence changes when exposed to specific chemicals.
	Colorimetric sensors		Gas/liquid	∼0.1 ppm	Detect gases by causing color changes in chemically reactive dyes.
	Infrared (IR) gas sensors		Gas	∼0.1 ppm	Detect gases by measuring IR absorption at specific wavelengths.
**Other**	Capacitive sensors		Gas	∼1 ppm	Measure changes in capacitance caused by gas adsorption on the sensor surface.
	Thermal conductivity sensors		Gas	∼100 ppm	Detect gases by measuring changes in thermal conductivity of a sample compared to a reference gas.

Detection limit describes the smallest concentration detectable by the typical commercial sensor; specialized or lab-built alternatives may achieve lower limits than those listed.

These sensor technologies are integrated into both custom-built arrays and commercially available eNose systems, such as the Cyranose 320, aeoNose, PEN3, and SpiroNose (Table S1).

While some sensor arrays include multiple sensor types, most use a single sensor type but incorporate multiple sensor models, each with different chemical selectivities, ie, that respond to different sets of chemicals.

## Methods

### Search strategy

To compile the studies included in this review, we conducted a search using PubMed and examined the bibliographies of existing literature. We did not limit our search by year.

### Inclusion and exclusion criteria

Exclusion criteria were applied as follows:

1. Studies that did not use a sensor array.
2. Studies that did not use unaltered and non-invasively extracted human samples, including studies that analyzed cell lines.
3. Studies that did not classify a cancer group against a non-cancer group.
4. Studies that employed compound separation techniques or used sensor arrays designed to identify specific compounds.
5. Studies that lacked sufficient methodological detail.
6. Studies that did not report empirical results for a final classification model.
7. Studies that analyzed samples in liquid form, ie, using an eTongue rather than an eNose.

### Review methodology

Each study was read by one of the authors, including the methodology and results. We synthesized each study by describing the cancer type studied; the type of sample analyzed (eg, breath or urine); the number and types of sensors in the eNose; the sizes of the training and test datasets and the type of model validation performed; the modeling techniques employed; and the results metrics.

### Treatment of multiple analyses

In cases where the cancer group was classified against different control groups, we report all classifications in separate rows. In cases where separate classifications were performed upon subgroups, such as smokers and non-smokers, we only include the full classification if one exists; otherwise, we report each subclassification in separate rows.

For studies that performed multiclass classification between cancer and two separate control groups, such as cancer patients vs. healthy controls vs. patients with benign diseases, we calculate the metrics after pooling the control groups.

### Training and test sets

The training set refers to any samples used during the model development at any point, including cross-validation sets. The test set refers to external samples not involved in model training or validation and only analyzed when the training process is completed.^47^ Sample sizes were determined based on these definitions regardless of the terminology employed in the study.

### Metrics

We report on three metrics:

1. Sensitivity, defined as True Positives/Total Positives.
2. Specificity, defined as True Negatives/Total Negatives.
3. Accuracy, defined as (True Positive + True Negatives)/(Total Positives + Total Negatives).

If a confusion matrix or misclassifications were listed in the text, we calculated these metrics directly. Otherwise, we used the reported values.

### Model methods

In the *Model Methods* column, we detail the feature extraction and classification techniques used. If an ensemble was used, all constituent models are listed. When studies tested multiple methods, we report only the method highlighted in the abstract or the one with the highest accuracy.

### Sensor array

The *Sensor Array* column describes the device utilized in the study. We only list sensors that directly detect VOCs and omit temperature and RH sensors, as well as sensors placed outside the main detection chamber to monitor external conditions. For commercial devices, we name the device directly.

### Quality assessment

To evaluate the methodological quality of the included studies, we used the Quality Assessment of Diagnostic Accuracy Studies 2 (QUADAS-2) tool.^48^ Sensitivity analyses were conducted by re-running meta-regressions excluding studies with ≥2 domains rated as “High” risk of bias.

### Meta-analysis methodology

We conducted a meta-analysis on studies that included an independent test set. In cases where a study reported multiple independent classification analyses (eg, separate cancer types or distinct control groups), we split them into separate data points.

We performed three sets of analyses:

1. Descriptive visualizations.
2. Stratified meta-analyses by subgroup.
3. Bivariate meta-regressions with single moderators.

Both the stratified meta-analyses and meta-regressions utilized a bivariate random-effects model with restricted maximum likelihood (REML) estimation to predict sensitivity and specificity jointly while accounting for correlation.^49^^,^^50^ Proportions were logit-transformed prior to analysis.

For stratified meta-analyses, we grouped studies by categorical variables and estimated pooled sensitivity and specificity within each subgroup.

For meta-regressions, we modeled each moderator (categorical or numeric) as a covariate, including an interaction term with outcome type (sensitivity vs. specificity) to allow for differential effects. Between-study heterogeneity was captured using an unstructured covariance matrix.^51^ We assessed significance with the QM test^52^ and applied Benjamini–Hochberg false discovery rate (FDR) correction to adjust for multiple comparisons.^53^ Due to the limited total sample size, regressions with multiple covariates were not performed.

Categorical moderators with fewer than three data points per subgroup were excluded to reduce instability. For numeric moderators, we also visually inspected regression prediction plots for nonlinear trends or clusters, and excluded outliers based on multiple influence diagnostics: Cook’s distance, studentized residuals, and leverage.^54^

### Reporting standards

This process abided by the Preferred Reporting Items for Systematic Reviews and Meta-Analyses (PRISMA) guidelines.^55^ This review was registered on the Open Science Framework (OSF) registry (DOI: https://doi.org/10.17605/OSF.IO/MHBWC).

## Results

### Overview

After removing duplicates, 1 041 studies were initially identified, 825 studies were removed during screening, 3 studies could not be accessed, and 109 studies were excluded based on the eligibility criteria, leaving 104 studies for inclusion in this review. Of those, 37 studies included independent test sets and were thus included in the meta-analysis. (See Figure S1 for the PRISMA flowchart summarizing this process.)

Table 2 provides a summary of the reviewed papers included in the meta-analysis; additional studies included in the systematic review can be found in Table S2. Studies have been organized by cancer type and year.

**Table 2.** Summary of papers included in the meta-analysis.

Cancer type	Paper name	Sample type	Sensor array	Classification problem	Test group (train)	Control group (train)	Model val. methods	Test group (test)	Control group (test)	Model methods	Sens.	Spec.	Acc.
**Lung**	Machado et al. (2005, United States)^56^	Breath	Cyranose 320	Lung cancer vs. healthy + benign diseases	14	20 + 25	Unspec.	14	32 + 30	SVM	71.4%	91.9%	88.2%
**Lung**	Mazzone et al. (2007, United States)^57^	Breath	Col. (36 spots)	Lung cancer vs. non-cancer	100	15		43	6	RF	73.3%	72.4%	73.2%
**Lung**	Hubers et al. (2014, Netherlands)^58^	Breath	Cyranose 320	Lung cancer vs. COPD	20	31		18	8	PCA, ROC	94.4%	12.5%	69.2%
**Lung**	Gasparri et al. (2016, Italy)^59^	Breath	8 QCM	Lung cancer vs. healthy	51	54	LOOCV	21	20	PLS-DA	81.0%	100%	90.2%
**Lung**	Tirzīte et al. (2017, Latvia)^60^	Breath	Cyranose 320	Lung cancer vs. healthy	120	63	Unspec.	45	16	SVM	97.8%	68.8%	90.2%
				**Lung cancer vs. non-cancer**	120	131	Unspec.	45	39	SVM	88.9%	66.7%	78.6%
**Lung**	Huang et al. (2018, Taiwan)^61^	Breath	Cyranose 320	Lung cancer vs. non-cancer	44	159	8:2 ×N	12	29	SVM, LDA	83.3%	86.2%	85.4%
**Lung**	Van de Goor et al. (2018, Netherlands)^62^	Breath	aeoNose	Lung cancer vs. benign conditions	52	93	10%-out CV	8	14	ANN	87.5%	85.7%	86.4%
**Lung**	Kononov et al. (2020, Russia)^63^	Breath	6 MOS	Lung cancer vs. healthy	45	37	5-fold CV	20	16	PCA, kNN, SVM	95.0%	100%	97.2%
**Lung**	Gharra et al. (2020, Israel)^64^	Breath	15 GNP	Lung cancer vs. healthy	156	106	LOOCV	66	47	DFA	100%	100%	100%
**Lung**	Chen et al. (2020, China)^65^	Breath	8 rGO	Lung cancer vs. healthy	23	24		24	25	ANN	95.8%	96.0%	95.9%
**Lung**	Binson et al. (2021, India)^66^	Breath	5 MOS	Lung cancer vs. healthy	41	74		10	19	KPCA, XGBoost	70.0%	84.2%	79.3%
**Lung**	Binson et al. (2021, India)^67^	Breath	5 MOS	Lung cancer vs. healthy + COPD	32	72 + 38	3/5/10-fold CV	8	18 + 10	PCA, kNN	75.0%	89.3%	86.1%
**Lung**	Binson and Subramoniam (2021, India)^68^	Breath	5 MOS	Lung cancer vs. healthy	15	22		12	17	PCA, SVM	89.6%	88.2%	88.8%
**Lung**	Van de Sar et al. (2023, Netherlands)^69^	Breath	SpiroNose	Lung cancer vs. ILD	31	37		15	18	PLS-DA	100%	89%	94%
				**Lung cancer vs. COPD**	31	34		15	16	PLS-DA	93%	100%	97%
				**Lung cancer vs. IPF**	31	41		15	20	PLS-DA	87%	100%	94%
**Lung**	Kort et al. (2023, Netherlands)^70^	Breath	aeoNose	NSCLC vs. non-cancer	160	216	10%-out CV	76	120	ANN, LR, RF, modified RF, XGBoost	94.7%	49.2%	67.3%
**Lung**	Hao and Guang (2023, China)^71^	Breath	3 Amp., 1 MOS	Lung cancer vs. healthy	91	51	8:2 split ×100	12	10	GA, SVM, kNN, RF, LR, LDA, ANN, stacking	92.5%	92.2%	92.4%
**Lung**	Lee et al. (2024, Taiwan)^72^	Breath	14 MOS	Lung cancer vs. healthy + benign diseases	90	16 + 62	7:3 split	28	10 + 25	SSDG, NSA, CNN	82.1%	68.6%	74.6%
**Lung**	Binson et al. (2024, India)^73^	Breath	5 MOS	Lung cancer vs. healthy	72	96	10-fold CV	42	51	KPCA, XGBoost	83.3%	86.3%	84.9%
**Lung**	Chen et al. (2024, Taiwan)^74^	Breath	Cyranose 320	Lung cancer vs. healthy	89	35	3:1 split	22	9	SMOTE, kNN	88%	100%	94%
**Lung**	Buma et al. (2025, Netherlands)^75^	Breath	SpiroNose	Lung cancer vs. other cancers and diseases				216	148	PCA, LDA	94.9%	50.7%	76.9%
				**Lung cancer vs. other cancers and diseases**	143	100		72	49	LR	94.4%	63.3%	81.8%
**Gastric**	Xu et al. (2013, Israel)^76^	Breath	10 GNP, 4 CNT	Gastric cancer vs. benign conditions	31	67	LOOCV	6	26	DFA	83.3%	96.2%	93.8%
**Gastric**	Amal et al. (2016, Israel)^77^	Breath	5 GNP, 1 CNT	Gastric cancer vs. healthy + GIM	69	230	LOOCV	30	95	DFA	73.3%	97.9%	92.0%
			**3 GNP**	Gastric cancer vs. PUD	69	38	LOOCV	30	15	DFA	86.7%	86.7%	86.7%
**Gastric**	Broza et al. (2019, Israel)^78^	Breath	6 GNP	Gastric cancer vs. non-cancer	99	342	LOOCV	3	723	DFA	100%	78.8%	78.9%
**Gastric**	Gharra et al. (2020, Israel)^64^	Breath	15 GNP	Gastric cancer vs. healthy	117	109	LOOCV	53	44	DFA	100%	97.7%	99.0%
**Gastric**	Leja et al. (2021, Latvia)^79^	Breath	8 GNP	Gastric cancer vs. non-cancer	31	65		16	40	LDA	100%	87.5%	91.1%
**Ovarian**	Amal et al. (2016, Israel)^80^	Breath	1 GNP, 2 CNT	Ovarian cancer vs. healthy	34	34	LOOCV	14	14	DFA	78.6%	100%	89.3%
			**4 GNP, 1 CNT**	Ovarian cancer vs. benign tumors	34	61	LOOCV	14	25	DFA	57.1%	60.0%	59.0%
			**2 GNP, 2 CNT**	Ovarian cancer vs. healthy + benign tumors	34	34 + 61	LOOCV	14	14 + 25	DFA	71.4%	71.8%	71.7%
**Ovarian**	Raspagliesi et al. (2020, Italy)^81^	Breath	PEN3	Ovarian cancer vs. healthy	58	76	5-fold CV	28	38	PCA, kNN	100%	100%	100%
				**Ovarian cancer vs. healthy + benign masses**	58	76 + 34	5-fold CV	28	38 + 17	PCA, kNN	100%	100%	100%
**Prostate**	Bax et al. (2022, Italy)^82^	Urine	6 MOS	Prostate cancer vs. healthy	59	24	5-fold CV	22	17	OSC, Boruta, RF	81.8%	70.6%	76.9%
**Prostate**	Taverna et al. (2022, Italy)^26^	Urine	6 MOS	Prostate cancer vs. healthy + other cancers and diseases				88	28 + 58	OSC, Boruta, RF	85.2%	79.1%	82.2%
**Prostate**	Talens et al. (2023, Spain)^83^	Urine	32 MOS	Prostate cancer vs. BPH	10	10		10	10	ANN	89.8%	83.7%	86.7%
**Colo-rectal**	Amal et al. (2016, Israel)^84^	Breath	1 GNP, 2 CNT	Colorectal cancer vs. healthy	45	86	LOOCV	20	36	DFA	85.0%	94.4%	91.1%
**Colo-rectal**	Van Keulen et al. (2019, Netherlands)^85^	Breath	aeoNose	Colorectal cancer vs. healthy	42	68	10%-out CV	20	36	ANN	80.0%	64.0%	69.7%
**Colo-rectal**	Poļaka et al. (2023, Latvia)^86^	Breath	56 MOS and GNP	Colorectal cancer vs. non-cancer	73	130		32	56	GA, RF	53.3%	93.0%	78.4%
**Breast**	Barash et al. (2015, Israel)^87^	Breath	40 GNP and CNT	Breast cancer vs. healthy + benign conditions	64	20 + 10	LOOCV	32	10 + 5	DFA	84%	80%	83%
**Breast**	Benet et al. (2022, United States)^88^	Urine	4 MOS	Breast cancer vs. healthy	18	18	4 samples set aside	2	2	CNN	100%	50.0%	75.0%
**Meso-thel ioma**	Chapman et al. (2011, Australia)^89^	Breath	Cyranose 320	MPM vs. healthy	10	10	Unspec.	10	32	PCA, CDA	90.0%	90.6%	90.5%
**Head and Neck**	Mohamed et al. (2021, Norway)^90^	Breath	aeoNose	OSCC vs. healthy	49	35	10%-out CV	10	17	ANN	80.0%	76.5%	77.8%
**Bladder**	Jian et al. (2022, China)^91^	Urine	10 Poly.	Bladder cancer vs. healthy	52	12	3-fold CV	24	6	RFE, SVM	100%	83.3%	96.7%

Each row lists the cancer type and classification task, the sample type and sensor array, group sizes for training and test sets, modeling and validation methods, and reported sensitivity, specificity, and accuracy. Studies with multiple analyses appear in separate rows.

Abbreviations:

Sensor array: Amp., amperometric sensor; CNT, carbon nanotube sensor; Col., colorimetric sensor; GNP, graphene nanoplatelet sensor; MOS, metal-oxide semiconductor sensor; Poly., polymer-based sensor; QCM, quartz crystal microbalance sensor; rGO, reduced graphene oxide sensor.

Classification problem: BPH, benign prostatic hyperplasia; COPD, chronic obstructive pulmonary disease; GIM, gastric intestinal metaplasia; ILD, interstitial lung disease; IPF, idiopathic pulmonary fibrosis; MPM, malignant pleural mesothelioma; NSCLC, non-small cell lung cancer; OSCC, oral squamous cell carcinoma; PUD, peptic ulcer disease.

Model validation methods: *K*-fold CV, *K*-fold cross-validation; *K1: K2* split ×*N*, train/test split with ratio *K1: K2*, repeated *N* times; *X*%-out CV, leave-*X*%-out cross-validation; LOOCV, leave-one-out cross-validation; Unspec., validation used but type unspecified.

Model methods: ANN, artificial neural network; Boruta, Boruta feature selection; CDA, canonical discriminant analysis; CNN, convolutional neural network; DFA, discriminant function analysis; GA, genetic algorithm; kNN, k-nearest neighbors; KPCA, kernel principal component analysis; LDA, linear discriminant analysis; LR, logistic regression; NSA, noise-shift augmentation; OSC, orthogonal signal correction; PCA, principal component analysis; PLS-DA, partial least squares discriminant analysis; RF, random forest; RFE, recursive feature elimination; ROC, receiver operating characteristic curve analysis; SMOTE, synthetic minority oversampling technique; SSDG, semi-supervised domain generalization; SVM, support vector machine; XGBoost, extreme gradient boosting.

Several of the 37 studies included more than one independent classification analysis (eg, different cancer types, sample types, or control groups), resulting in 46 total data points used in the meta-analysis. The total sample sizes were 1 365 for the cancer groups and 2 249 for the control groups. Across the 37 studies, there was a wide range of false positive and false negative results (Table S3). What is to be noted, however, is that the resultant sensitivities and specificities were generally good.

Across all 46 data points, the pooled estimates from the meta-analysis were:

- Sensitivity: 85.9% (95% CI: 82.3-88.9%)
- Specificity: 83.6% (95% CI: 78.6-87.7%)

**Figure 2.**
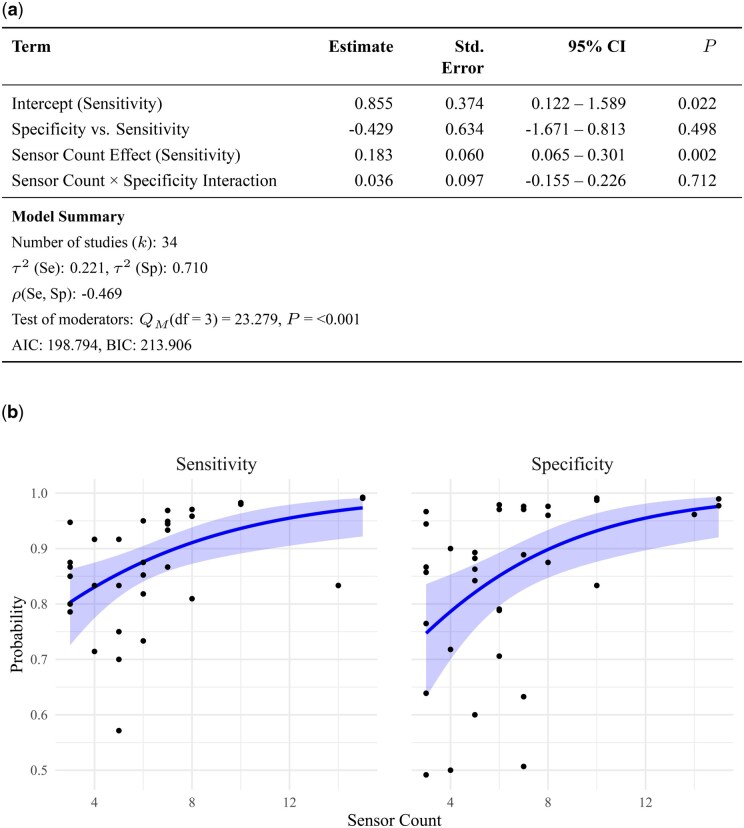
Sensor count meta-regression (devices with ≤15 sensors). (a) Meta-regression coefficients and model fit statistics. (b) Predicted sensitivity and specificity by sensor count with 95% confidence interval bands.

Exploratory analyses are shown in Figure S2. Pooled metrics from stratified analyses are presented by cancer type (Figure 2), sample type (Figure S3), and sensor type (Figure S4). The meta-regression results are summarized in Table 3. Results for individual studies are presented in Table S3 and Figure S7.

**Table 3.** Summary of meta-regression models.

Moderator Variable	Type	*k*	*Q_M_* (df)	*P*	*P*_FDR_	*τ^2^* (Se)	*τ^2^* (Sp)	*ρ* (Se, Sp)
**Cancer Type**	Cat.	41	11.26 (9)	.259	0.465	0.300	0.964	-0.306
**Sample Type**	Cat.	46	1.64 (3)	.650	0.731	0.372	0.824	-0.277
**Sensor Count (all)**	Num.	46	2.79 (3)	.425	0.547	0.292	0.830	-0.314
**Sensor Count (≤15 sensors)**	Num.	34	23.28 (3)	<.001	<0.001	0.221	0.710	-0.469
**Sensor Type**	Cat.	43	4.98 (5)	.419	0.547	0.399	0.815	-0.226
**Sensor Array Type**	Cat.	42	15.23 (9)	.085	0.191	0.259	0.809	-0.065

Each model estimates the association of a single moderator with logit-transformed sensitivity and specificity using bivariate random-effects meta-regression. Reported values include the QM statistic (*Q_M_*), FDR-adjusted *P* value, between-study variances (*τ^2^*), and correlation (*ρ*) between sensitivity and specificity.

### Cancer type

Lung cancer was the most studied type, represented in 20 of the 37 studies. Studies of lung cancer had a pooled sensitivity of 87.3% (95% CI: 83.1-90.6%) and specificity of 80.4% (95% CI: 72.8-86.3%). The next most commonly studied type, gastric cancer, achieved a sensitivity of 86.9% (95% CI: 75.6-93.4%) and specificity of 91.7% (95% CI: 81.8-96.5%), followed by ovarian cancer with a sensitivity of 86.4% (95% CI: 59.9-96.4%) and specificity of 91.6% (95% CI: 63.8-98.5%). All cancer types studied achieved pooled sensitivities and specificities above 70%, with most around 85% (Figure 2).

Bivariate meta-regression on cancer type (*k *= 41) found no significant difference in sensitivity and specificity between cancer types (*Q_M_*(df = 9) = 11.26, *P*_FDR_ = 0.47).

### Sample type

The majority of studies analyzed breath samples (32 studies), and the rest urine (5). Pooled sensitivity and specificity were similar across sample types: 86.0% (95% CI: 82.1-89.2%) and 84.9% (95% CI: 79.5-89.1%) for breath, and 85.7% (95% CI: 78.6-90.7%) and 77.5% (95% CI: 69.1-84.1%) for urine. Bivariate meta-regression (*k *= 47) found no significant difference by sample type (*Q_M_*(df = 3) = 1.64, *P*_FDR_ = 0.73).

### Sensor count

The number of sensors per device ranged from 3 to 56, with a mean of 13.5 and standard deviation of 13.1.

Bivariate meta-regression across all studies (*k *= 46) found no significant association between sensor count and either sensitivity or specificity (*Q_M_*(df = 3) = 2.79, *P*_FDR_ = 0.55). However, the prediction plot (Figure S5) revealed two distinct clusters: one with 3-15 sensors showing a visible positive trend, and another starting at 32 sensors (primarily using the Cyranose 320), where no relationship was apparent.

To investigate further, we repeated the meta-regression using only studies with ≤15 sensors. One extreme outlier (Lee et al.^72^) was excluded based on influence diagnostics. The final model (*k * = 34) showed a significant association (*Q_M_*(df = 3) = 23.28, *P*_FDR_ < 0.001), with improved model fit relative to the full dataset (AIC = 198.8 vs. 288.3; BIC = 213.9 vs. 305.6). Between-study heterogeneity was moderate for sensitivity (τ^2^ = 0.221) and higher for specificity (τ^2^ = 0.710), with a moderate negative correlation between them (ρ = -0.469).

Sensor count was significantly associated with sensitivity: Each additional sensor in the sensor array increased the logit-transformed sensitivity by 0.183 (95% CI: 0.065–0.301, *P *< .01). No significant effect was observed for specificity (between-measure term: *P *= .50; interaction term: *P *= .71).

### Sensor type

Six sensor types were represented across the dataset. However, only the three most common types—MOS (22 analyses), nanomaterial-based sensors (13), and polymer-based sensors (8)—had sufficient representation to be included in the meta-analysis. Bivariate meta-regression (*k *= 43) showed no significant association between sensor type and sensitivity or specificity (*Q_M_*(df = 5) = 4.98, *P*_FDR_ = 0.55).

### Quality assessment

QUADAS-2 assessments are summarized in Table S4 and Figure S6. The most common high-risk domain was *Patient Selection*, with 63.0% of analyses rated as “High.” Sensitivity analyses of meta-regressions, in which studies with ≥2 “High” risk of bias ratings were excluded, did not yield materially different results.

## Discussion

### Cancer type and sample type

eNose technology proved applicable across a broad range of cancer types, with all types yielding pooled sensitivities and specificities above 70% and most around 85%. This suggests that, despite the nascent state of this area of research, there is strong potential for this technology—and even the same device—to develop into a screening tool for numerous cancer types.

Lung cancer dominates the literature. Several authors cited as motivation the limitations of the current non-invasive diagnostic standard, low-dose computed tomography (LDCT).^93^^,^^94^ While LDCT has relatively high sensitivity (93.5%) and specificity (73.4%), it is costly, and its high false-positive rate (PPV = 3.8%) often leads to unnecessary follow-up procedures.^95^^,^^96^ Given that lung cancer is the leading cause of cancer-related death,^97^ a low-cost screening test with similar or better diagnostic performance to LDCT could have significant public health impact.

Cancer type is often tracked with sample type: all lung cancer studies used breath, and all prostate cancer and bladder cancer studies used urine. This pattern likely reflects the assumption that sample types anatomically closer to the tumor site may yield stronger or more specific VOC signals. That assumption may be reasonable for bladder cancer, where the tumor typically forms on the inner lining in direct contact with stored urine, and possibly for lung cancer, where most tumors are located near the airways.

In general, cancer alters cellular metabolism, producing VOCs that enter the bloodstream and are exhaled or excreted. This systemic mechanism implies that any sample type can carry a cancer-specific VOC fingerprint, regardless of tumor location. In support of this, Mohamed et al.^98^ analyzed lung cancer using urine, breath, and blood samples and found very similar results across all three sample types. In a similar vein, our meta-analysis found no significant difference in sensitivity or specificity between breath and urine samples. These findings suggest that both sample types should be considered viable for VOC-based cancer detection, regardless of cancer type.

### Sensor count

Among studies using sensor arrays with 15 or fewer sensors, our meta-regression found a significant positive relationship between the number of sensors and sensitivity. This suggests that increasing the sensor count enhances detection performance—likely by improving the system’s capability in capturing complex VOC patterns. Because the model was estimated on the logit scale, effect size varies depending on baseline sensitivity. For illustration, increasing from 9 to 10 sensors raises the predicted sensitivity by 1.18 points, and from 10 to 11 by 1.01 points (Figure 2B).

### Sensor type

Sensor type was not significantly associated with sensitivity or specificity in the meta-regression. However, this analysis was limited to only three sensor categories—metal-oxide semiconductor (MOS), nanomaterial-based, and polymer-based—due to insufficient representation of other types. Notably, all three fall under the broader class of chemiresistive sensors, which constrains the generalizability of this finding.

Of the studies reviewed, 35 of 37 (94.6%) included chemiresistive sensors in their sensor array, likely due to their low cost and wide availability.^99^ Because the role of the sensor array is to detect a VOC “fingerprint” rather than to identify individual compounds, more precise but costlier sensors such as QCM or infrared sensors may offer little advantage and may undermine the economic feasibility of deploying eNoses in clinical settings.

A key drawback, however, of chemiresistive sensors is the risk of sensor drift, the gradual decline in sensor accuracy over time, unlike more stable mass-based sensors.^99^ Bax et al.,^82^ who utilized MOS sensors, noted that sensor drift poses the “primary obstacle to the [eNose] diffusion for long-term applications.”

While many studies employed basic processing methods—eg baseline correction, normalization, scaling, replicate averaging^56^^,^^61^—only three applied more advanced techniques. Bax et al.^82^ and Taverna et al.^26^ applied orthogonal signal correction (OSC) and Lee et al.^72^ applied semi-supervised domain generalization (SSDG) and noise-shift augmentation (NSA); all three reported improved performance and reduced drift. Broader testing and adoption of these techniques or others, such as periodic recalibration or other domain adaptation algorithms, will be necessary before eNose systems can be considered viable for widespread clinical use.

### Study design and sample sizes

A major constraint in this body of research is the relatively small sample sizes. Across the 46 analyses, the average number of patients used for model training was only 120.7. This limits the application of deep neural networks, which often require hundreds or thousands of samples to perform reliably. Neural networks—which are particularly well-suited for detecting complex VOC patterns—were used in only 11 of the 37 studies (29.7%).

Only 37 of the 104 (35.6%) papers identified tested their models on a held-out test set, with an average test dataset size of just 78.6. These small test sets weaken the strength of validation and are another significant limitation of our meta-analysis.

Moreover, 31 of the 37 studies included in the meta-analysis utilized a case-control study design, with separate, often non-random, recruitment of cancer patients and control subjects. Four studies employed prospective recruitment—ie, the enrollment of study participants before cancer status is known (eg, symptomatic patients or population-based cohorts)—a design which more closely reflects real-world scenarios. Two studies used a mixed approach. For this reason, the majority of analyses were assessed as exhibiting “High” risk of bias in the *Patient Selection* domain. This bias should be avoided by enrolling a representative clinical population consecutively or randomly at the point of care, prior to diagnostic confirmation.

These considerations reflect the experimental nature of the field: Studies are often early-stage feasibility efforts, piloting custom-built sensor arrays or new analytical pipelines. While these studies help demonstrate proof-of-concept, larger datasets and prospective study designs are needed for more rigorous validation and to advance eNose technology to clinical use.

### Methodological variation

Studies exhibited wide methodological variation, much of which was not captured in our summary tables or meta-analyses.

Patient instructions varied. Some studies specified collecting early morning samples, when VOC concentration is generally higher and not influenced by recent food consumption.^81^^,^^91^ For this reason, about half the studies also instructed participants to fast for varying amounts of time before sample collection.^59^^,^^71^ In studies using breath samples, participants were sometimes instructed not to smoke, use mouthwash, wear perfumes, drink coffee or alcohol, or use medications before collection.^43^^,^^58^^,^^66^

Sample collection protocols differed across multiple dimensions. Some studies analyzed fresh samples;^63^^,^^92^ others froze urine samples^88^^,^^91^ or adsorbed breath samples^78^^,^^87^ for later analysis. Sampling bags and sorbent tubes for breath samples also varied in type and brand.

Analytical procedures for sample preparation and measurement were equally diverse. Urine samples were analyzed at room temperature in some studies,^91^ while others heated samples to various temperatures.^26^^,^^92^ For breath, all studies analyzed samples at room temperature, except for Mazzone et al.,^57^ who incubated the samples at body temperature. Some studies also specified maintaining a constant relative humidity in the sensing chamber.^73^^,^^82^

Headspace preparation techniques differed in implementation. Some studies circulated air or an inert gas over the sample to push VOCs into the sensor chamber,^57^^,^^81^ while others had patients breathe continuously into the eNose device.^62^^,^^86^ In certain cases, VOCs were first trapped on adsorbent materials and later released via thermal desorption, allowing pre-concentration of trace compounds.^64^^,^^78^ Specific methods varied in terms of carrier gas, timing, flow rate, desorption temperature, and sensor integration.

Studies varied in methods for data preprocessing, feature extraction, and classification techniques, as noted in the summary table. Furthermore, among the 104 studies reviewed overall, six studies^70^^,^^75^^,^^94^^,^^100–102^ tested their classification models with and without the inclusion of clinical parameters, five of which found that including these variables increased the performance of their model relative to using eNose data alone. This suggests that other studies could improve their results by including them, and that future studies should seek to secure additional data that could be used alongside sensor data for classification.

### Evaluation of methodologies

A small number of studies tested multiple methodologies explicitly: Capelli et al.^27^ compared conditioning temperatures for urine samples at 23 °C, 37 °C, 50 °C, and 60 °C, finding that 60 °C yielded the best classification performance without risking protein denaturation. Asimakopoulos et al.^103^ and Capelli et al.^27^ tested multiple portions of the urine stream and found that the initial portion improved detection efficacy. However, neither of these studies employed independent test sets. While these comparative studies are valuable, by and large, more studies are needed to compare methodologies and help standardize these approaches.

There have not been a significant amount of studies on what factors affect sensitivity and specificity. Amal et al.^84^ assessed the effect of overnight fasting on the performance of their model and found no difference, indicating that fasting may not improve results. Research is needed to determine whether abstention from smoking or alcohol or other test conditions alter these metrics. In addition, it is unknown how oncologic interventions—eg, surgery or chemotherapy—prior to the eNose test affect results.

### Limitations

This meta-analysis had several limitations. First, as discussed above, relatively few studies used independent test sets; those that did typically had small sample sizes, limiting the strength of our meta-analyses. Second, substantial methodological heterogeneity across studies—including differences in sample collection, preparation, and analysis—introduces confounding that could not be controlled given the limited number of studies in each subgroup. Third, in order to preserve statistical power, we included multiple data points from studies that conducted several classification tasks, which may have introduced cross-sample correlation. Fourth, our meta-analysis does not distinguish between (1) different subtypes of a cancer, (2) whether healthy individuals or patients with benign conditions were used as controls, and (3) differences in patient recruitment, which could introduce selection bias.

## Conclusion

This review highlights both the promise and limitations of eNose technology in cancer detection. Despite wide methodological variability, high pooled sensitivity, and specificity were found across cancer types, suggesting strong potential for future non-invasive diagnostics.

Our meta-analysis found that increasing the number of sensors—up to 15—was associated with improved model sensitivity. However, other meta-regressions yielded non-significant results.

Advancing eNose technology will require several key improvements: standardization of sampling and analysis protocols, robust sensor drift compensation, integration of clinical variables, larger, more diverse datasets, and prospective study design. Ultimately, as the field matures, rigorous validation, and methodologic standardization will be essential to move eNoses from experimental tools to clinically reliable diagnostics with the potential to transform early cancer detection.

Despite the advantages of this technology that we have highlighted in this review, a lack of widespread familiarity persists. We hope this publication will make the cancer research community more aware of the potential for eNose technology.

## Supplementary Material

oyag016_Supplementary_Data

## Data Availability

The extracted data and analysis code used in this meta-analysis from the corresponding author may be provided upon reasonable request.
