# Supplementary material for: eNose technologies in the detection of cancer: a systematic review and meta-analysis
Source: Oncologist. 2026 Jan 28;31(3):oyag016. doi: 10.1093/oncolo/oyag016 (PMC12923108; doi:10.1093/oncolo/oyag016)
Supplement: oyag016_Supplementary_Data [file oyag016_supplementary_data.pdf]

Supplementary Table S1. Commercially Available eNoses Used in Medical Research

Product Name	Sensor Array	Description
aeoNose	3 MOS	Used in over 20 studies, primarily for detection of lung and colon cancers. Operations have been suspended due to lack of funding.[1]
Cyranose 320	32 Polymer-based	Used in over 250 studies for detection of various diseases including cancer, COVID-19, and respiratory conditions. Connects to PCnose software for custom “Smellprint” library. Widespread use in food, industrial, and manufacturing applications.[2]
PEN3	10 MOS	Used in only a handful of studies for disease detection. Primarily used for quality control in the food, chemical, and pharmaceutical industries.[3]
SpiroNose	28 MOS	Cloud-calibrated, linked to BreathBase for data analysis. Used in over 30 studies for detection of lung cancer, respiratory conditions, and other diseases. 14 MOS sensors are externally placed for environmental monitoring. (Some studies use an older model with fewer sensors.)[4]

Supplementary Table S2. Summary of Papers Included in the Narrative Review
Each row lists the cancer type and classification task, the sample type and sensor array, group sizes for training sets, modeling and validation methods, and reported sensitivity, specificity, and accuracy. Studies with multiple analyses appear in separate rows.

Cancer Type	Paper Name	Sample Type	Sensor Array	Classification Problem	Test Group (Train)	Control Group (Train)	Model Val. Methods	Model Methods	Sens.	Spec.	Acc.
Lung	Di Natale et al. (2003, Italy) [5]	Breath	8 QCM	Lung cancer vs. healthy + DF survivors	35	18+9	LOOCV	PLS-DA	100%	100%	100%
Lung	Blatt et al. (2007, Italy) [6]	Breath	6 MOS	Lung cancer vs. healthy	43	58	LOOCV	Non-Parametric LDA, Fuzzy kNN	95.3%	90.5%	92.6%
Lung	D'Amico et al. (2010, Italy) [7]	Breath	8 QCM	Lung cancer vs. healthy	28	36	LOOCV	PLS-DA	85.7%	100%	93.8%
				Lung cancer vs. benign diseases	28	28	LOOCV	PLS-DA	92.9%	78.6%	85.7%
Lung	Hakim et al. (2011, Israel) [8]	Breath	5 GNP	Lung cancer vs. healthy	20	26	2-fold CV	PCA, SVM	100%	92.3%	95.7%
Lung	Wang et al. (2012, China) [9]	Breath	9 MOS	Lung cancer vs. healthy	47	42	LOOCV	ANN	74.5%	69.0%	71.9%
Lung	Broza et al. (2012, Israel) [10]	Breath	4 GNP, 2 PtNP	Lung cancer vs. benign nodules	24	10	LOOCV	DFA	100%	80.0%	94.1%
Lung	Mazzone et al. (2012, United States) [11]	Breath	Col. (24 spots)	NSCLC vs. non-cancer	83	137	Boot. ×N	LR	70.0%	86.0%	80.0%
				SCLC vs. non-cancer	9	137	Boot. ×N	LR	89.0%	85.0%	85.2%
Lung	Santonico et al. (2012, Italy) [12]	Breath	8 QCM	Lung cancer vs. benign tracheal stenosis	20	10	LOOCV	PLS-DA	81.0%	62.0%	75.0%
Lung	Peled et al. (2013, Israel) [13]	Breath	16 GNP, 2 CNT	Lung cancer vs. benign nodules	49	19	LOOCV	DFA	86.0%	96.0%	88.0%
Lung	Bikov et al. (2014, Hungary) [14]	Breath	Cyranose 320	Lung cancer vs. healthy	27	37	LOOCV	PCA, CDA	81.0%	76.0%	78.0%
Lung	Mazzone et al. (2015, United States) [15]	Urine	Col. (73 spots)	Lung cancer vs. benign nodules + at risk	90	24+31	7:3 split ×100	RF	81.4%	60.0%	73.3%
				NSCLC vs. benign nodules + at risk	84	24+31	7:3 split ×100	RF	80.0%	64.7%	73.9%
Lung	McWilliams et al. (2015, Canada) [16]	Breath	Cyranose 320	Lung cancer vs. high-risk non-cancer	25	166	10-fold CV	DT	88.0%	81.3%	82.2%
Lung	Rocco et al. (2015, Italy) [17]	Breath	7 QCM	Lung cancer vs. healthy	23	77	LOOCV	PLS-DA	82.6%	96.1%	93.0%
Lung	Tan et al. (2016, Malaysia) [18]	Breath	1 Chemires.	Lung cancer vs. healthy + COPD	12	13+12		ROC	83.3%	88.0%	86.5%
Lung	Li et al. (2017, China) [19]	Breath	9 MOS, 4 Amp., 2 CC	Lung cancer vs. healthy	24	23	10-fold CV	LDA, Fuzzy kNN	91.6%	91.7%	91.6%
Lung	Shlomi et al. (2017, Israel) [20]	Breath	26 GNP, 14 CNT	Early lung cancer vs. benign nodules	16	30	LOOCV	DFA	75.0%	93.3%	87.0%
Lung	Chang et al. (2018, South Korea) [21]	Breath	7 MOS	NSCLC vs. healthy	37	48	2-fold CV	PCA, ANN	79.0%	72.0%	75.0%
Lung	Kort et al. (2018, Netherlands) [22]	Breath	aeoNose	NSCLC vs. non-cancer	144	146	10%-out CV	ANN	94.4%	32.9%	63.4%
				SCLC vs. non-cancer	18	75	10%-out CV	ANN	88.9%	80.0%	81.7%
Lung	Tirzīte et al. (2018, Latvia) [23]	Breath	Cyranose 320	Lung cancer vs. non-cancer (smokers)	119	91		LR	95.8%	92.3%	94.3%
				Lung cancer vs. non-cancer (non-smokers)	133	132		LR	96.2%	90.9%	93.6%
Lung	Lu et al. (2019, China) [24]	Breath	8 MOS, 2 Amp., 1 CC	Lung cancer vs. healthy	98	116	7:2:1 split ×50	GRU-AE, DT, MSEP, VC	94.2%	92.8%	93.6%
Lung	Marzorati et al. (2019, Italy) [25]	Breath	4 MOS	Lung cancer vs. healthy	6	10	LOOCV	ANN	85.7%	100%	93.8%
Lung	Mohamed et al. (2019, Egypt) [26]	Blood	PEN3	Lung cancer vs. healthy	26	21	60:25:15 split	PCA, ANN	96.2%	95.2%	95.7%
		Breath	PEN3	Lung cancer vs. healthy	28	20	60:25:15 split	PCA, ANN	92.9%	90.0%	91.7%
		Urine	PEN3	Lung cancer vs. healthy	27	24	60:25:15 split	PCA, ANN	100%	100%	100%
Lung	Fielding et al. (2020, Australia) [27]	Breath	Cyranose 320	Bronchial SCC vs. non-cancer	10	13	LOOCV	RF	77.0%	80.0%	72.0%
Lung	Kort et al. (2020, Netherlands) [28]	Breath	aeoNose	NSCLC vs. non-cancer	138	143	Boot. ×1000	ANN, LR	95.7%	59.7%	77.4%

Cancer Type	Paper Name	Sample Type	Sensor Array	Classification Problem	Test Group (Train)	Control Group (Train)	Model Val. Methods	Model Methods	Sens.	Spec.	Acc.
Lung	Krauss et al. (2020, Germany) [29]	Breath	aeoNose	Lung cancer vs. healthy	91	33	10%-out CV	ANN	84.0%	97.0%	87.5%
Lung	Rodríguez-Aguilar et al. (2021, Mexico) [30]	Breath	Cyranose 320	Lung cancer vs. healthy	30	50	LOOCV	PCA, CAP	100%	100%	100%
Lung	Binson et al. (2021, India) [31]	Breath	5 MOS, 1 Amp., 2 CC	Lung cancer vs. healthy	48	63	3/5/10-fold CV	KPCA, XGBoost	90.6%	92.7%	91.7%
				Lung cancer vs. COPD	48	52	3/5/10-fold CV	ICA, XGBoost	81.4%	88.1%	84.8%
Lung	Binson et al. (2021, India) [32]	Breath	5 MOS	Lung cancer vs. COPD	32	38	3/5/10-fold CV	PCA, kNN	78.1%	81.6%	80.0%
Lung	Binson et al. (2021, India) [33]	Breath	5 MOS	Lung cancer vs. healthy	32	72	3/5/10-fold CV	PCA, kNN	84.4%	94.4%	91.3%
Lung	Chen et al. (2021, China) [34]	Breath	7 MOS, 2 Amp., 2 CC	Lung cancer vs. non-cancer	101	134	10-fold CV	KPCA, XGBoost	95.6%	91.1%	93.6%
				Lung cancer vs. COPD	33	28	10-fold CV	KPCA, XGBoost	95.0%	96.7%	96.0%
Lung	Liu et al. (2021, China) [35]	Breath	7 MOS, 2 Amp., 2 CC	Lung cancer vs. non-cancer	98	116	10-fold CV	PCA, LR, SVM, kNN, RF, DT, VC	95.3%	97.2%	96.1%
Lung	Gasparri et al. (2022, Italy) [36]	Urine	12 QCM, 1 PID	NSCLC vs. healthy	46	81	7:3 split ×100	SVM	72.7%	70.8%	71.6%
Head and Neck	Hakim et al. (2011, Israel) [8]	Breath	5 GNP	HNSCC vs. healthy	16	26	2-fold CV	PCA, SVM	100%	92.3%	95.2%
Head and Neck	Gruber et al. (2014, Israel) [37]	Breath	5 GNP, 1 CNT	HNSCC vs. healthy	22	19	LOOCV	DFA	77.0%	90.0%	83.0%
				HNSCC vs. benign tumors	22	21	LOOCV	DFA	77.0%	90.0%	84.0%
Head and Neck	Leunis et al. (2014, Netherlands) [38]	Breath	12 MOS	HNSCC vs. benign conditions	36	23	Boot. ×N	LR with forward selection	91.7%	82.6%	88.1%
Head and Neck	Van de Goor et al. (2019, Netherlands) [39]	Breath	aeoNose	HNSCC vs. DF survivors	20	20	10%-out CV	ANN	85.0%	80.0%	82.5%
Head and Neck	Fielding et al. (2020, Australia) [27]	Breath	Cyranose 320	Laryngeal HNSCC vs. non-cancer	12	13	LOOCV	RF	100%	85.0%	92.0%
Head and Neck	Van de Goor et al. (2020, Netherlands) [40]	Breath	aeoNose	HNSCC vs. healthy	91	72	10%-out CV	TD, ANN	79.1%	62.5%	71.8%
Head and Neck	Anzivino et al. (2022, Italy) [41]	Breath	Cyranose 320	HNSCC vs. healthy + AR	15	15+15	LOOCV	PCA, CDA	93.3%	86.7%	88.9%
Bladder	Weber et al. (2010, United Kingdom) [42]	Urine	17 MOS, 1 IR	Bladder TCC vs. healthy + benign conditions	30	20+39	LOOCV	PLS-DA	60.0%	66.9%	64.6%
Bladder	Heers et al. (2018, Germany) [43]	Urine	Cyranose 320	Bladder TCC vs. healthy	30	30	K-fold CV	LDA	93.3%	93.3%	93.3%
Bladder	Matsumoto et al. (2020, Japan) [44]	Urine	2 MOS	Bladder cancer vs. healthy	36	27		ROC	61.4%	52.8%	57.7%
Bladder	Zhu et al. (2020, United Kingdom) [45]	Urine	Fluo. (24 spots)	Bladder cancer vs. healthy	38	41	LOOCV; 8:2 split ×100	PLS-DA	77.4%	85.8%	81.8%
Bladder	Bassi et al. (2021, Italy) [46]	Urine	Cyranose 320	Bladder cancer vs. benign conditions	102	96		LDA	91.2%	89.6%	90.4%
Breast	Shuster et al. (2010, Israel) [47]	Breath	1 GNP, 1 PtNP	Breast cancer vs. healthy + benign conditions	13	7+16	K-out CV	SVM	84.6%	95.7%	91.7%
Breast	Barash et al. (2015, Israel) [48]	Breath	40 GNP and CNT	Breast cancer vs. benign conditions	169	52	LOOCV	DFA	71.1%	79.0%	76.6%
Breast	Herman-Saffar et al. (2018, Israel) [49]	Breath	12 MOS	Breast cancer vs. healthy	33	32	K-fold CV	ANN	89.2%	100%	94.3%
			Cyranose 320	Breast cancer vs. healthy	33	32	K-fold CV	ANN	91.7%	78.3%	84.5%
Breast	Díaz de León-Martínez et al. (2020, Mexico) [50]	Breath	Cyranose 320	Breast cancer vs. healthy	262	181	LOOCV, K-fold CV	PCA, CDA	100%	100%	100%
Breast	Rodríguez-Aguilar et al. (2021, Mexico) [30]	Breath	Cyranose 320	Breast cancer vs. healthy	50	50	LOOCV	PCA, CAP	100%	100%	100%
Prostate	Asimakopoulos et al. (2014, Italy) [51]	Urine	8 QCM	Prostate cancer vs. BPH	14	27	LOOCV	PLS-DA	71.4%	92.6%	85.4%
Prostate	Waltman et al. (2018, Netherlands) [52]	Breath	aeoNose	Prostate cancer vs. healthy + BPH	32	30+23	10-fold CV	ANN	84.0%	70.0%	75.0%
Prostate	Bax et al. (2021, Italy) [53]	Urine	3 MOS	Prostate cancer vs. healthy	78	37	5-fold CV	RF	79.5%	86.5%	81.7%

Cancer Type	Paper Name	Sample Type	Sensor Array	Classification Problem	Test Group (Train)	Control Group (Train)	Model Val. Methods	Model Methods	Sens.	Spec.	Acc.
Prostate	Capelli et al. (2021, Italy) [54]	Urine	6 MOS	Prostate cancer vs. healthy	132	60	10-fold CV	Boruta, RF	81.8%	86.7%	83.3%
Breast	Yang et al. (2021, Taiwan) [55]	Breath	Cyranose 320	Breast cancer vs. healthy	351	88	LOOCV	RF	86.0%	97.0%	91.0%
Prostate	Filianoti et al. (2022, Italy) [56]	Urine	Cyranose 320	Prostate cancer vs. healthy	133	139	LOOCV	PCA, CDA	82.7%	88.5%	85.7%
Prostate	Heers et al. (2024, Germany) [57]	Urine	Cyranose 320	Prostate cancer vs. healthy	56	53	LOOCV	PCA, LDA	76.8%	62.3%	69.7%
Colo-rectal	De Meij et al. (2014, Italy) [58]	Feces	Cyranose 320	Colorectal cancer vs. healthy + AA	40	57+60	1000-fold MC CV	PCA, CDA	85.0%	87.0%	86.5%
Colo-rectal	Westenbrink et al. (2015, United Kingdom) [59]	Urine	8 Amp., 2 IR, 1 PID	Colorectal cancer vs. IBS	39	35	LOOCV	LDA	78.0%	79.0%	78.5%
Colo-rectal	Altomare et al. (2016, Italy) [60]	Breath	PEN3	Colorectal cancer vs. healthy + benign polyps	15	15+15	LOOCV	PCA, PNN	93.3%	10.0%	37.8%
Colo-rectal	Amal et al. (2016, Israel) [61]	Breath	1 GNP	Colorectal cancer vs. ADEN	16	16	LOOCV	DFA	93.8%	87.5%	90.6%
Colo-rectal	Tyagi et al. (2021, United Kingdom) [62]	Urine	PEN3	Colorectal cancer vs. non-cancer	58	38	10-fold CV	ANN	91.4%	81.6%	87.5%
Gastric	Daniel & Thangavel (2016, India) [63]	Breath	3 MOS	Gastric cancer vs. healthy + gastric ulcers + benign conditions	49	82+19 +11	10-fold CV	ANN	94.4%	89.9%	93.0%
Gastric	Schuermans et al. (2018, Netherlands) [64]	Breath	aeoNose	Gastric cancer vs. healthy	16	28	LOOCV	ANN	81.2%	71.4%	75.0%
Gastric	Poļaka et al. (2022, Latvia) [65]	Breath	18 MOS, 8 GNP	Gastric cancer vs. healthy	54	85	8:2 split ×1000	Information Gain, NB	64.0%	85.0%	77.8%
Meso-thelioma	Dragonieri et al. (2011, Italy) [66]	Breath	Cyranose 320	MPM vs. healthy	13	13	LOOCV	PCA, CDA	92.3%	92.3%	92.3%
				MPM vs. asbestos-exposed	13	13	LOOCV	PCA, CDA	100%	84.6%	92.3%
Leukemia	Bordbar et al. (2021, Iran) [67]	Blood	Col. (16 spots)	Leukemia vs. healthy (<18 years old)	59	47		PCA, LDA	98.3%	95.7%	97.2%
Leukemia	Baudrexler et al. (2023, Germany) [68]	Serum	Cyranose 320	AML vs. healthy	17	14		LDA	82.4%	78.6%	80.6%
Chondro-sarcoma	Evenhuis et al. (2024, Netherlands) [69]	Breath	aeoNose	Chondrosarcoma vs. healthy	24	25	10%-out CV	ANN	75.0%	64.0%	69.4%
				Chondrosarcoma vs. healthy + benign lesions	24	25+8	10%-out CV	ANN	75.0%	63.6%	68.4%
Cervical	Díaz De León-Martínez et al. (2021, Mexico) [70]	Urine	Cyranose 320	Cervical cancer vs. healthy	12	12	LOOCV	CAP	91.7%	100%	95.8%
Renal	Murdocca et al. (2021, Italy) [71]	Urine	12 QCM	ccRCC vs. healthy	30	25	7:3 split ×100	LDA	100%	83.3%	91.7%
Renal	Costantini et al. (2023, Italy) [72]	Urine	Cyranose 320	Renal cancer vs. healthy	110	142	LOOCV	PCA, CDA	71.8%	88.7%	81.3%
Thyroid	Scheepers et al. (2022, Netherlands) [73]	Breath	aeoNose	Thyroid cancer vs. benign diseases	48	85	10%-out CV	RF, LR	93.8%	95.3%	94.7%
Ovarian	Angioli et al. (2024, Italy) [74]	Breath	7 QCM	Ovarian cancer vs. benign masses	50	43	LOOCV	PLS-DA	82.0%	76.7%	79.6%

Abbreviations.
Sensor Array: Amp. = Amperometric Sensor; CC = Catalytic Combustion Sensor; Chemires. = Chemiresistor; CNT = Carbon Nanotube Sensor; Col. = Colorimetric Sensor; Fluo. = Fluorometric Sensor; GNP = Graphene Nanoplatelet Sensor; IR = Infrared Sensor; MOS = Metal-Oxide Semiconductor Sensor; PID = Photoionization Detector; Pot. = Potentiometric Sensor; PtNP = Platinum Nanoparticle Sensor; QCM = Quartz Crystal Microbalance Sensor
Classification Problem: AA = Advanced Adenoma; ADEN = Adenoma; AML = Acute Myeloid Leukemia; AR = Allergic Rhinitis; BPH = Benign Prostatic Hyperplasia; ccRCC = Clear Cell Renal Cell Carcinoma; COPD = Chronic Obstructive Pulmonary Disease; DF = Disease-free; HNSCC = Head and Neck Squamous Cell Carcinoma; IBS = Irritable Bowel Syndrome; MPM = Malignant Pleural Mesothelioma; NSCLC = Non-Small Cell Lung Cancer; OSCC = Oral Squamous Cell Carcinoma; SCC = Squamous Cell Carcinoma; SCLC = Small Cell Lung Cancer; TCC = Transitional Cell Carcinoma
Model Validation Methods: K-fold CV = K-fold cross-validation; K-out CV = Leave-K-out cross-validation; $K_1 : K_2$ split $\times N$ = Train/test split with ratio $K_1 : K_2$, repeated N times; $K_1 : K_2 : K_3$ split = Train/validation/test split; $X\%$-out CV = Leave-$X\%$-Out Cross-Validation; Boot. $\times N$ = Bootstrap resampling over N iterations; LOOCV = Leave-One-Out Cross-Validation; MC CV = Monte Carlo Cross-Validation
Model Methods: ANN = Artificial Neural Network; Boruta = Boruta Feature Selection; CAP = Canonical Analysis of Principal Coordinates; CDA = Canonical Discriminant Analysis; DFA = Discriminant Function Analysis; DT = Decision Trees; GRU-AE = Gated Recurrent Unit Autoencoder; ICA = Independent Component Analysis; kNN = k-Nearest Neighbors; KPCA = Kernel Principal Component Analysis; LDA = Linear Discriminant Analysis; LR = Logistic Regression; MSEP = Novel Ensemble Pruning Method [20]; NB = Naive Bayes; PCA = Principal Component Analysis; PLS-DA = Partial Least Squares Discriminant Analysis; PNN = Probabilistic Neural Network; RF = Random Forest; ROC = Receiver Operating Characteristic Curve Analysis; SVM = Support Vector Machine; TD = Tucker Decomposition; VC = Voting Classifier; XGBoost = Extreme Gradient Boosting

Supplementary Table S3. Per-Study Performance Summary

Paper Name	Classification Problem	TP	FN	FP	TN	Pooled Sens.	Sens. (95% CI)	Pooled Spec.	Spec. (95% CI)
Machado et al. (2005, United States)	Lung cancer vs. healthy + benign diseases	10	4	5	57	0.714	0.419 – 0.916	0.919	0.822 – 0.973
Mazzone et al. (2007, United States)	Lung cancer vs. non-cancer	32	11	2	4	0.733	0.588 – 0.865	0.724	0.223 – 0.957
Hubers et al. (2014, Netherlands)	Lung cancer vs. COPD	17	1	7	1	0.944	0.727 – 0.999	0.125	0.003 – 0.527
Gasparri et al. (2016, Italy)	Lung cancer vs. healthy	17	4	0	20	0.810	0.581 – 0.946	1.000	0.832 – 1.000
Tirzīte et al. (2017, Latvia)	Lung cancer vs. healthy	44	1	5	11	0.978	0.882 – 0.999	0.688	0.413 – 0.890
Tirzīte et al. (2017, Latvia)	Lung cancer vs. non-cancer	40	5	13	26	0.889	0.759 – 0.963	0.667	0.498 – 0.809
Huang et al. (2018, Taiwan)	Lung cancer vs. non-cancer	10	2	4	25	0.833	0.516 – 0.979	0.862	0.683 – 0.961
Van de Goor et al. (2018, Netherlands)	Lung cancer vs. benign conditions	7	1	2	12	0.875	0.473 – 0.997	0.857	0.572 – 0.982
Kononov et al. (2020, Russia)	Lung cancer vs. healthy	19	1	0	16	0.950	0.751 – 0.999	1.000	0.794 – 1.000
Gharra et al. (2020, Israel)	Lung cancer vs. healthy	66	0	0	47	1.000	0.946 – 1.000	1.000	0.925 – 1.000
Chen et al. (2020, China)	Lung cancer vs. healthy	23	1	1	24	0.958	0.789 – 0.999	0.960	0.796 – 0.999
Binson et al. (2021, India)	Lung cancer vs. healthy	7	3	3	16	0.700	0.348 – 0.933	0.842	0.604 – 0.966
Binson et al. (2021, India)	Lung cancer vs. healthy + COPD	6	2	3	25	0.750	0.349 – 0.968	0.893	0.718 – 0.977
Binson & Subramoniam (2021, India)	Lung cancer vs. healthy	11	1	2	15	0.896	0.615 – 0.998	0.882	0.636 – 0.985
Van de Sar et al. (2023, Netherlands)	Lung cancer vs. ILD	15	0	2	16	1.000	0.782 – 1.000	0.890	0.653 – 0.986
Van de Sar et al. (2023, Netherlands)	Lung cancer vs. COPD	14	1	0	16	0.930	0.681 – 0.998	1.000	0.794 – 1.000
Van de Sar et al. (2023, Netherlands)	Lung cancer vs. IPF	13	2	0	20	0.870	0.595 – 0.983	1.000	0.832 – 1.000
Kort et al. (2023, Netherlands)	NSCLC vs. non-cancer	72	4	61	59	0.947	0.871 – 0.985	0.492	0.399 – 0.584
Hao & Guang (2023, China)	Lung cancer vs. healthy	11	1	1	9	0.925	0.615 – 0.998	0.922	0.555 – 0.997
Lee et al. (2024, Taiwan)	Lung cancer vs. healthy + benign diseases	23	5	11	24	0.821	0.631 – 0.939	0.686	0.507 – 0.831
Binson et al. (2024, India)	Lung cancer vs. healthy	35	7	7	44	0.833	0.686 – 0.930	0.863	0.737 – 0.943
Chen et al. (2024, Taiwan)	Lung cancer vs. healthy	19	3	0	9	0.880	0.651 – 0.971	1.000	0.664 – 1.000
Buma et al. (2025, Netherlands)	Lung cancer vs. other cancers and diseases (a)	205	11	73	75	0.949	0.911 – 0.974	0.507	0.423 – 0.590
Buma et al. (2025, Netherlands)	Lung cancer vs. other cancers and diseases (b)	68	4	18	31	0.944	0.864 – 0.985	0.633	0.483 – 0.766
Xu et al. (2013, Israel)	Gastric cancer vs. benign conditions	5	1	1	25	0.833	0.359 – 0.996	0.962	0.804 – 0.999
Amal et al. (2016, Israel)	Gastric cancer vs. healthy + GIM	22	8	2	93	0.733	0.541 – 0.877	0.979	0.926 – 0.997
Amal et al. (2016, Israel)	Gastric cancer vs. PUD	26	4	2	13	0.867	0.693 – 0.962	0.867	0.595 – 0.983
Broza et al. (2019, Israel)	Gastric cancer vs. non-cancer	3	0	153	570	1.000	0.292 – 1.000	0.788	0.757 – 0.818
Gharra et al. (2020, Israel)	Gastric cancer vs. healthy	53	0	1	43	1.000	0.933 – 1.000	0.977	0.880 – 0.999
Leja et al. (2021, Latvia)	Gastric cancer vs. non-cancer	16	0	5	35	1.000	0.794 – 1.000	0.875	0.732 – 0.958
Amal et al. (2016, Israel)	Ovarian cancer vs. healthy	11	3	0	14	0.786	0.492 – 0.953	1.000	0.768 – 1.000
Amal et al. (2016, Israel)	Ovarian cancer vs. benign tumors	8	6	10	15	0.571	0.289 – 0.823	0.600	0.387 – 0.789
Amal et al. (2016, Israel)	Ovarian cancer vs. healthy + benign tumors	10	4	11	28	0.714	0.419 – 0.916	0.718	0.551 – 0.850
Raspagliesi et al. (2020, Italy)	Ovarian cancer vs. healthy	28	0	0	38	1.000	0.877 – 1.000	1.000	0.907 – 1.000
Raspagliesi et al. (2020, Italy)	Ovarian cancer vs. healthy + benign masses	28	0	0	55	1.000	0.877 – 1.000	1.000	0.935 – 1.000
Bax et al. (2022, Italy)	Prostate cancer vs. healthy	18	4	5	12	0.818	0.597 – 0.948	0.706	0.440 – 0.897

Paper Name	Classification Problem	TP	FN	FP	TN	Pooled Sens.	Sens. (95% CI)	Pooled Spec.	Spec. (95% CI)
Taverna et al. (2022, Italy)	Prostate cancer vs. healthy + other cancers and diseases	75	13	18	68	0.852	0.761 – 0.919	0.791	0.690 – 0.871
Talens et al. (2023, Spain)	Prostate cancer vs. BPH	9	1	2	8	0.898	0.555 – 0.997	0.837	0.444 – 0.975
Amal et al. (2016, Israel)	Colorectal cancer vs. healthy	17	3	2	34	0.850	0.621 – 0.968	0.944	0.813 – 0.993
Van Keulen et al. (2019, Netherlands)	Colorectal cancer vs. healthy	16	4	13	23	0.800	0.563 – 0.943	0.640	0.462 – 0.792
Poļaka et al. (2023, Latvia)	Colorectal cancer vs. non-cancer	17	15	4	52	0.533	0.347 – 0.709	0.930	0.827 – 0.980
Barash et al. (2015, Israel)	Breast cancer vs. healthy + benign conditions	27	5	3	12	0.840	0.672 – 0.947	0.800	0.519 – 0.957
Benet et al. (2022, United States)	Breast cancer vs. healthy	2	0	1	1	1.000	0.158 – 1.000	0.500	0.013 – 0.987
Chapman et al. (2011, Australia)	MPM vs. healthy	9	1	3	29	0.900	0.555 – 0.997	0.906	0.750 – 0.980
Mohamed et al. (2021, Norway)	OSCC vs. healthy	8	2	4	13	0.800	0.444 – 0.975	0.765	0.501 – 0.932
Jian et al. (2022, China)	Bladder cancer vs. healthy	24	0	1	5	1.000	0.858 – 1.000	0.833	0.359 – 0.996

Supplementary Table S4. Summary of QUADAS-2 Assessments
For each paper and classification problem, risk of bias and applicability concern is assessed as Low (green circle), High (red circle), or Unclear (yellow circle).

		Risk of Bias				Applicability Concern		
**Paper Name**	**Classification Problem**	**Patient Selection**	**Index Test**	**Reference Standard**	**Flow & Timing**	**Patient Selection**	**Index Test**	**Reference Standard**
Machado et al. (2005, United States) [75]	Lung cancer vs. healthy + benign diseases	🔴	🟢	🟢	🟢	🟢	🟢	🟢
Mazzone et al. (2007, United States) [76]	Lung cancer vs. non-cancer	🔴	🟡	🟢	🟢	🟢	🟢	🟢
Hubers et al. (2014, Netherlands) [77]	Lung cancer vs. COPD	🔴	🟢	🟢	🟢	🟢	🟢	🟢
Gasparri et al. (2016, Italy) [78]	Lung cancer vs. healthy	🔴	🟡	🟢	🟢	🔴	🔴	🟢
Tirzīte et al. (2017, Latvia) [79]	Lung cancer vs. healthy	🟡	🟢	🟢	🟡	🔴	🟢	🟢
Tirzīte et al. (2017, Latvia) [79]	Lung cancer vs. non-cancer	🟡	🟢	🟢	🟡	🟢	🟢	🟢
Huang et al. (2018, Taiwan) [80]	Lung cancer vs. non-cancer	🔴	🟡	🟢	🟢	🔴	🟢	🟢
Van de Goor et al. (2018, Netherlands) [81]	Lung cancer vs. benign conditions	🔴	🟢	🟢	🟢	🟢	🟢	🟢
Kononov et al. (2020, Russia) [82]	Lung cancer vs. healthy	🔴	🟡	🟢	🟢	🔴	🟡	🟢
Gharra et al. (2020, Israel) [83]	Lung cancer vs. healthy	🔴	🟡	🟡	🟡	🔴	🔴	🟡
Chen et al. (2020, China) [84]	Lung cancer vs. healthy	🔴	🟡	🟡	🟡	🔴	🔴	🟡
Binson et al. (2021, India) [85]	Lung cancer vs. healthy	🔴	🟡	🟡	🟡	🔴	🔴	🟡
Binson et al. (2021, India) [85]	Lung cancer vs. healthy + COPD	🔴	🟡	🟡	🟡	🔴	🔴	🟡
Binson & Subramoniam (2021, India) [86]	Lung cancer vs. healthy	🔴	🟡	🟡	🟡	🔴	🔴	🟡
Van de Sar et al. (2023, Netherlands) [87]	Lung cancer vs. ILD	🟢	🟡	🟢	🟢	🟢	🟢	🟢
Van de Sar et al. (2023, Netherlands) [87]	Lung cancer vs. COPD	🟢	🟡	🟢	🟢	🟢	🟢	🟢
Van de Sar et al. (2023, Netherlands) [87]	Lung cancer vs. IPF	🟢	🟡	🟢	🟢	🟢	🟢	🟢
Kort et al. (2023, Netherlands) [88]	NSCLC vs. non-cancer	🟢	🟢	🟢	🟢	🟢	🟢	🟢
Hao & Guang (2023, China) [89]	Lung cancer vs. healthy	🟡	🟡	🟡	🟡	🔴	🟢	🟡
Lee et al. (2024, Taiwan) [90]	Lung cancer vs. healthy + benign diseases	🔴	🟡	🟢	🟢	🟢	🟢	🟢

Paper Name	Classification Problem	Risk of Bias				Applicability Concern		
		Patient Selection	Index Test	Reference Standard	Flow & Timing	Patient Selection	Index Test	Reference Standard
Binson et al. (2024, India) [91]	Lung cancer vs. healthy	🔴	🟡	🟡	🟡	🔴	🔴	🟡
Chen et al. (2024, Taiwan) [92]	Lung cancer vs. healthy	🔴	🟡	🟢	🟢	🔴	🟢	🟢
Buma et al. (2025, Netherlands) [93]	Lung cancer vs. other cancers and diseases [validation]	🟢	🟢	🟢	🟢	🟢	🟢	🟢
Buma et al. (2025, Netherlands) [93]	Lung cancer vs. other cancers and diseases [original]	🟢	🟢	🟢	🟢	🟢	🟢	🟢
Xu et al. (2013, Israel) [94]	Gastric cancer vs. benign conditions	🟢	🟡	🟢	🟢	🟢	🔴	🟢
Amal et al. (2016, Israel) [95]	Gastric cancer vs. healthy + GIM	🟡	🟢	🟢	🟢	🟢	🟢	🟢
Amal et al. (2016, Israel) [95]	Gastric cancer vs. PUD	🟡	🟢	🟢	🟢	🟢	🟢	🟢
Broza et al. (2019, Israel) [96]	Gastric cancer vs. non-cancer	🟢	🟢	🟢	🟢	🟢	🟢	🟢
Gharra et al. (2020, Israel) [83]	Gastric cancer vs. healthy	🔴	🟡	🟡	🟡	🔴	🔴	🟡
Leja et al. (2021, Latvia) [97]	Gastric cancer vs. non-cancer	🟡	🟡	🟢	🟢	🟢	🟡	🟢
Amal et al. (2016, Israel) [98]	Ovarian cancer vs. healthy	🔴	🟡	🟡	🟢	🔴	🔴	🟢
Amal et al. (2016, Israel) [98]	Ovarian cancer vs. benign tumors	🟢	🟡	🟢	🟢	🟢	🔴	🟢
Amal et al. (2016, Israel) [98]	Ovarian cancer vs. healthy + benign tumors	🔴	🟡	🟡	🟢	🟢	🔴	🟢
Raspagliesi et al. (2020, Italy) [99]	Ovarian cancer vs. healthy	🔴	🟡	🟢	🔴	🔴	🟢	🟢
Raspagliesi et al. (2020, Italy) [99]	Ovarian cancer vs. healthy + benign masses	🔴	🟡	🟢	🔴	🟢	🟢	🟢
Bax et al. (2022, Italy) [100]	Prostate cancer vs. healthy	🔴	🟡	🔴	🔴	🔴	🔴	🟢
Taverna et al. (2022, Italy) [101]	Prostate cancer vs. healthy + other cancers and diseases	🔴	🟢	🟢	🟢	🔴	🔴	🟢
Talens et al. (2023, Spain) [102]	Prostate cancer vs. BPH	🔴	🟡	🟡	🟡	🟢	🔴	🟡
Amal et al. (2016, Israel) [61]	Colorectal cancer vs. healthy	🔴	🟡	🟢	🟢	🟢	🔴	🟢
Van Keulen et al. (2019, Netherlands) [103]	Colorectal cancer vs. healthy	🟢	🟢	🟢	🟢	🟢	🟢	🟢
Poļaka et al. (2023, Latvia) [104]	Colorectal cancer vs. non-cancer	🟢	🟢	🟢	🟢	🟢	🔴	🟢
Barash et al. (2015, Israel) [48]	Breast cancer vs. healthy + benign conditions	🔴	🟢	🟢	🟡	🟢	🔴	🟢
Benet et al. (2022, United States) [105]	Breast cancer vs. healthy	🔴	🟡	🟢	🟡	🔴	🔴	🟢

Paper Name	Classification Problem	Risk of Bias				Applicability Concern		
		**Patient Selection**	**Index Test**	**Reference Standard**	**Flow & Timing**	**Patient Selection**	**Index Test**	**Reference Standard**
Chapman et al. (2011, Australia) [106]	MPM vs. healthy	🔴	🟢	🟢	🟢	🔴	🟢	🟢
Mohamed et al. (2021, Norway) [107]	OSCC vs. healthy	🔴	🟡	🟢	🟡	🔴	🟢	🟢
Jian et al. (2022, China) [108]	Bladder cancer vs. healthy	🔴	🟡	🟡	🟡	🔴	🔴	🟡

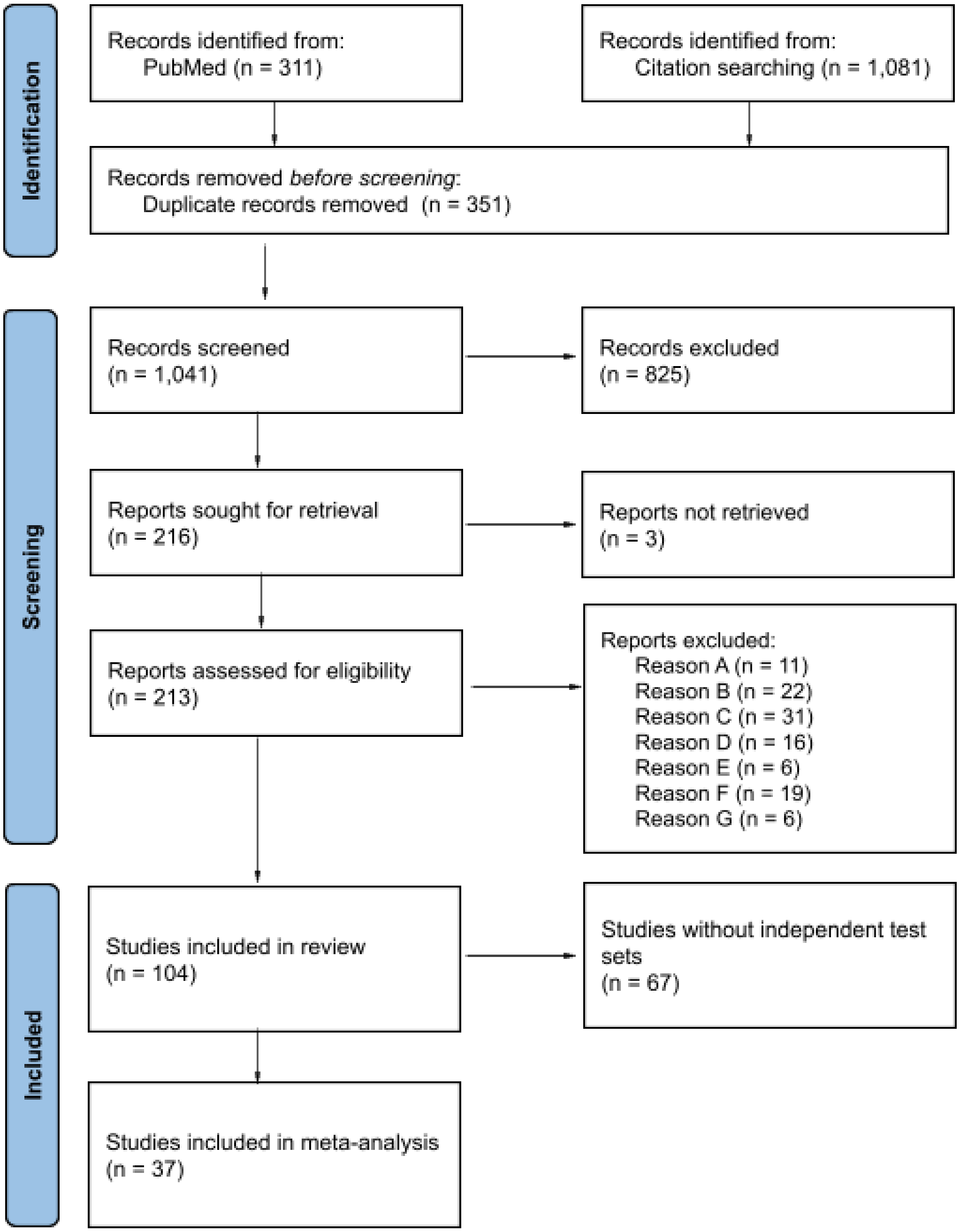

Supplementary Figure S1. PRISMA Diagram

Studies were excluded based on seven exclusion criteria: (A) absence of a sensor array; (B) failure to use unaltered and non-invasively extracted human samples; (C) no classification performed between cancer and non-cancer groups; (D) use of compound separation techniques; (E) insufficient methodological detail; (F) lack of final classification results; and (G) analysis of samples in liquid rather than gaseous form.

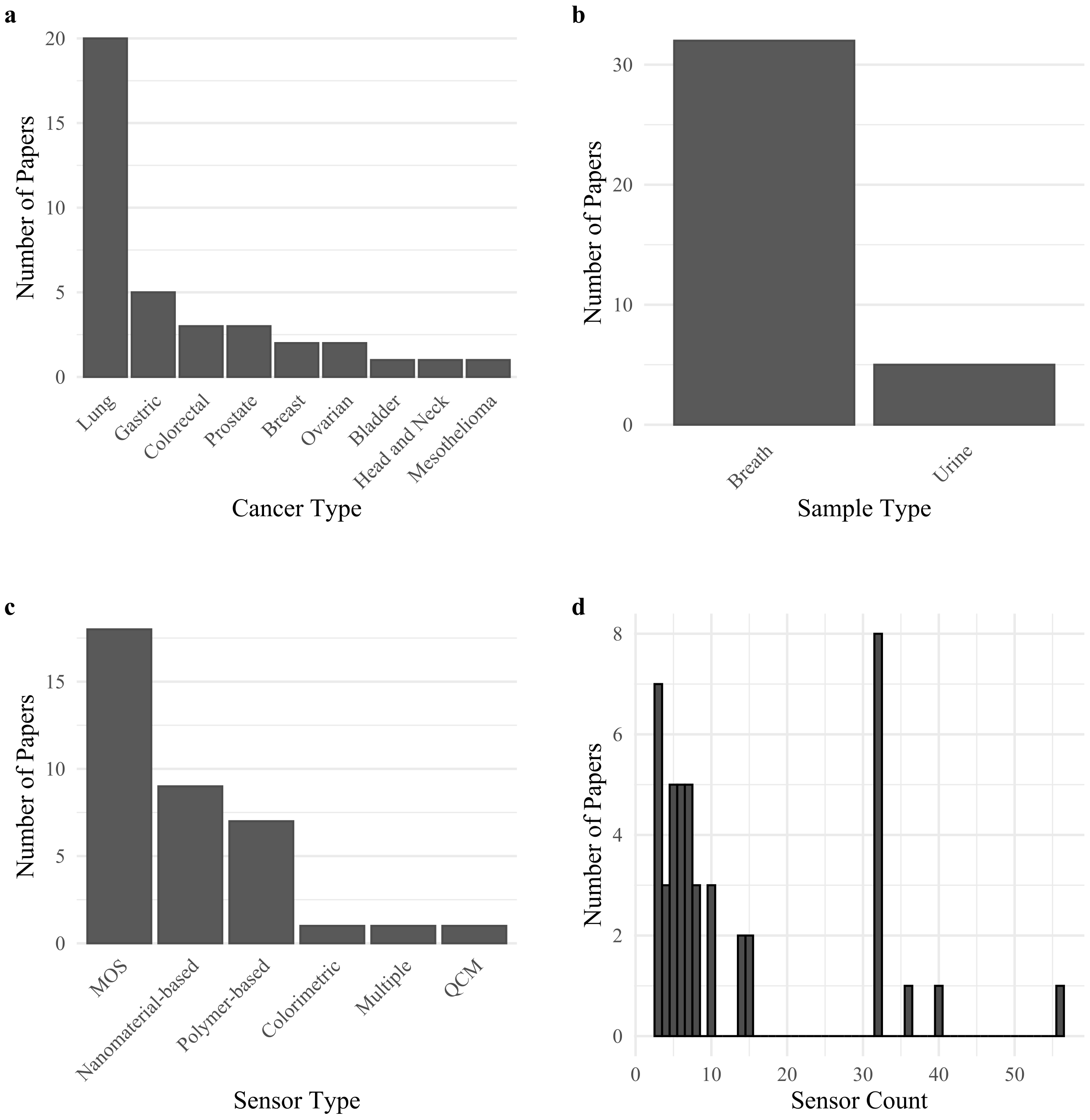

Supplementary Figure S2. Study Distribution by Category

a Bar chart by cancer type.

b Bar chart by sample type.

c Bar chart by sensor type.

d Histogram by sensor count.

a

Group	k	Pooled Sens.	Sens. SE	Sens. (95% CI)	Pooled Spec.	Spec. SE	Spec. (95% CI)
**All analyses**	46	0.859	0.138	0.823 – 0.889	0.836	0.168	0.786 – 0.877
Breath	41	0.860	0.152	0.821 – 0.892	0.849	0.190	0.795 – 0.891
Urine	5	0.857	0.249	0.786 – 0.907	0.775	0.220	0.691 – 0.841

b

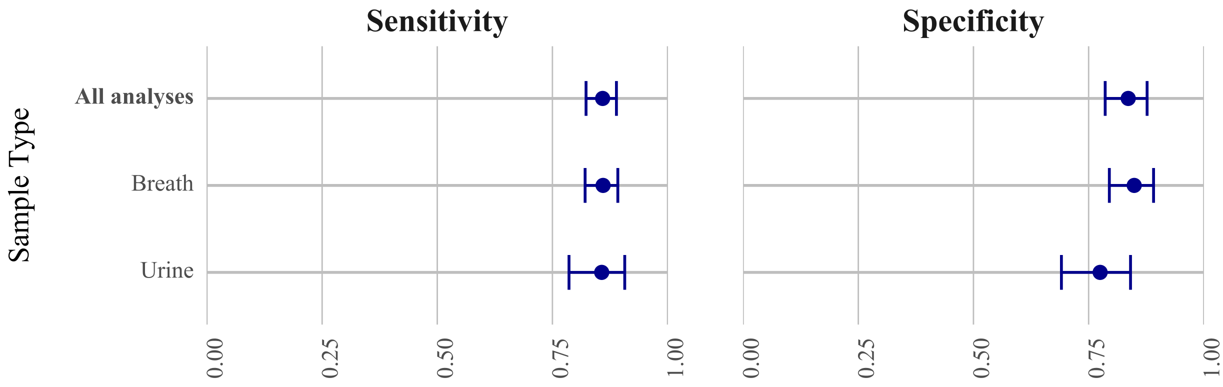

Supplementary Figure S3. Sample Type Stratified Meta-analysis
a Pooled sensitivity and specificity with 95% confidence intervals by sample type.
b Box-and-whisker plots showing pooled estimates with confidence intervals.

a

Group	k	Pooled Sens.	Sens. SE	Sens. (95% CI)	Pooled Spec.	Spec. SE	Spec. (95% CI)
**All analyses**	46	0.859	0.138	0.823 – 0.889	0.836	0.168	0.786 – 0.877
Polymer-based	8	0.886	0.332	0.802 – 0.937	0.785	0.424	0.614 – 0.893
MOS	22	0.855	0.192	0.801 – 0.895	0.782	0.195	0.710 – 0.840
Nanomaterial-based	13	0.847	0.296	0.756 – 0.908	0.903	0.356	0.823 – 0.949

b

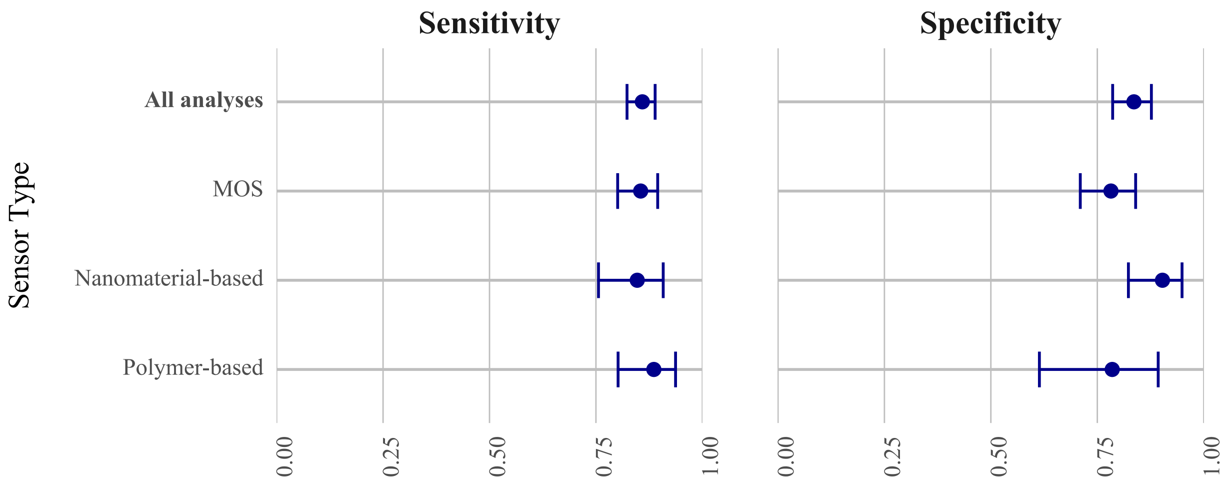

Supplementary Figure S4. Sensor Type Stratified Meta-analysis
a Pooled sensitivity and specificity with 95% confidence intervals by sensor type.
b Box-and-whisker plots showing pooled estimates with confidence intervals.

a

**Term**	**Estimate**	**Std. Error**	**95% CI**	*P*
Intercept (Sensitivity)	1.992	0.186	1.627 – 2.356	<0.001
Specificity vs. Sensitivity	-0.355	0.331	-1.004 – 0.293	0.283
Sensor Count Effect (Sensitivity)	-0.013	0.009	-0.031 – 0.004	0.130
Sensor Count × Specificity Interaction	0.014	0.017	-0.019 – 0.047	0.412

Model Summary

Number of studies (k): 46

τ^2 (Se): 0.292, τ^2 (Sp): 0.830

ρ(Se, Sp): -0.314

Test of moderators: $Q_M(\text{df} = 3) = 2.788$, $P = 0.425$

AIC: 288.291, BIC: 305.633

b

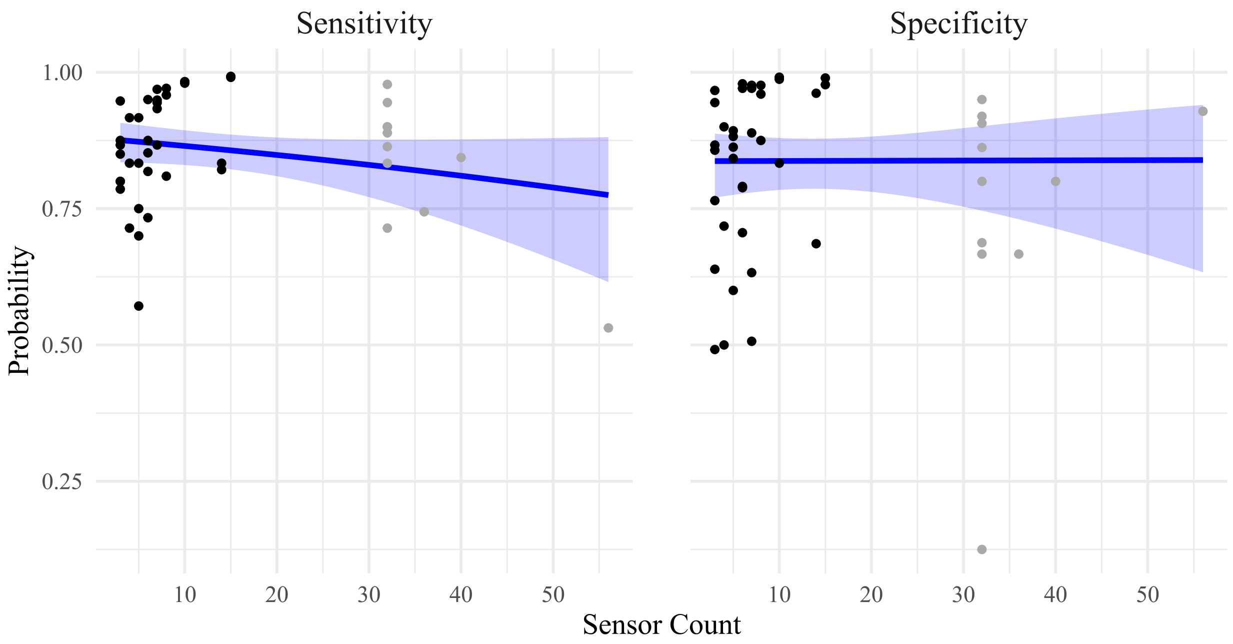

Supplementary Figure S5. Sensor Count Meta-regression (All Devices)
a Meta-regression coefficients and model fit statistics.
b Predicted sensitivity and specificity by sensor count with 95% confidence interval bands. Data points from the cluster of devices with ≤15 sensors are shown in black, and those from the cluster with ≥32 sensors are shown in gray.

a

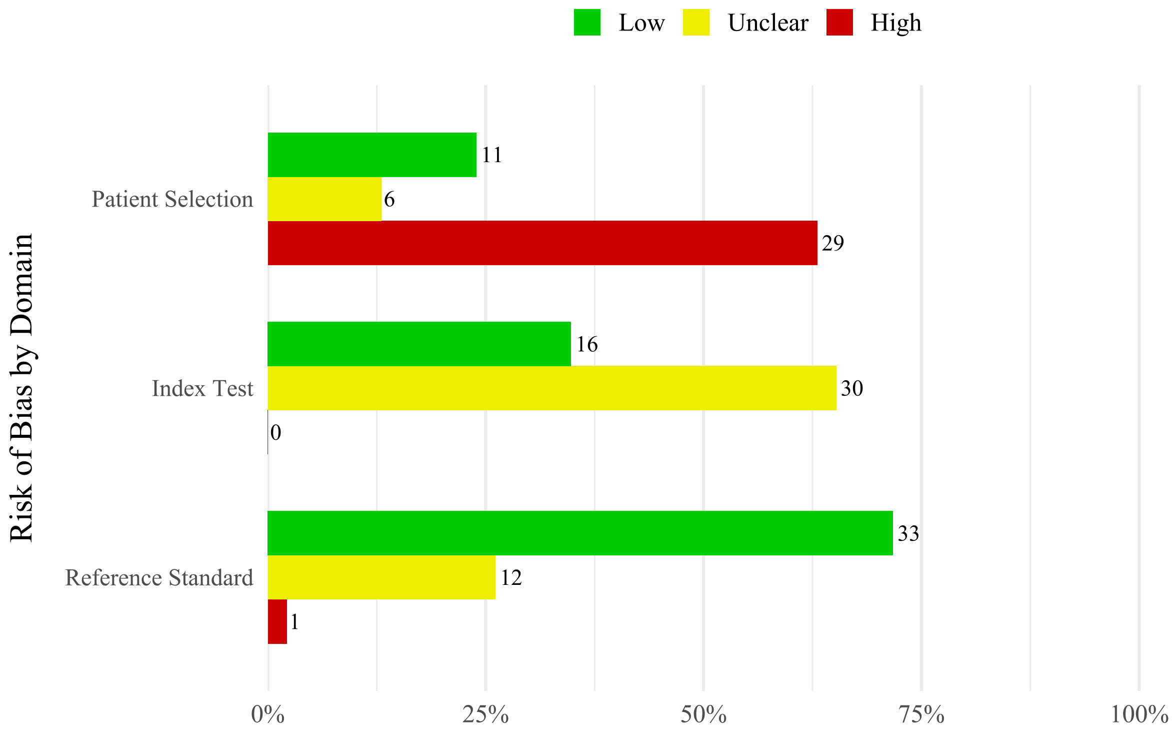

b

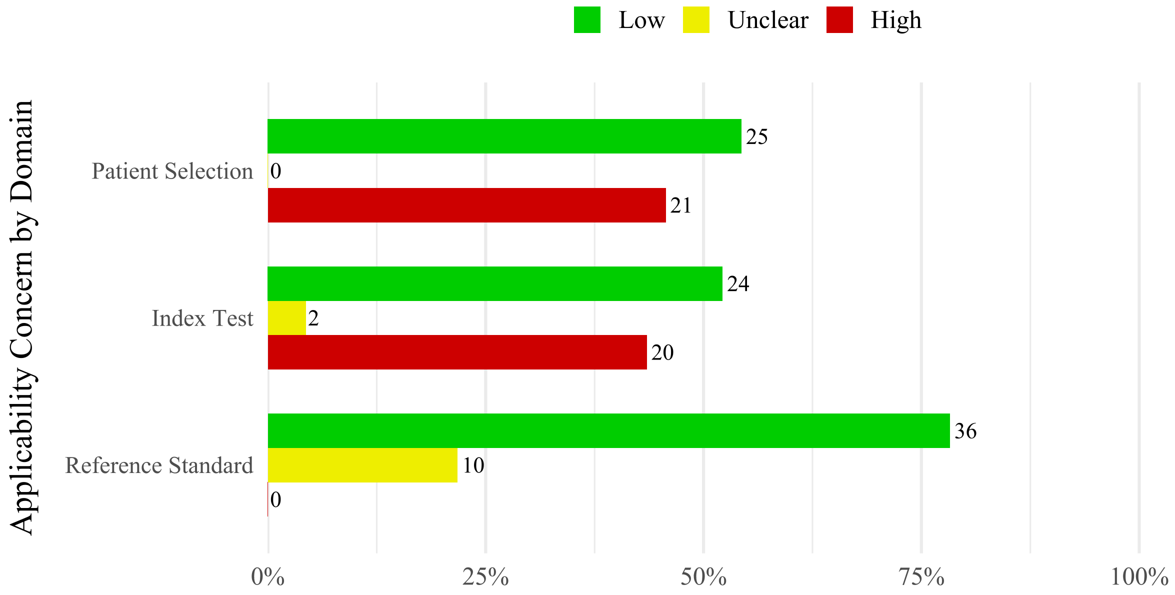

Supplementary Figure S6. QUADAS-2 Risk of Bias by Domains
a Bar plot showing the distribution of risk of bias judgments across domains.
b Bar plot showing the distribution of applicability concern judgments across domains.

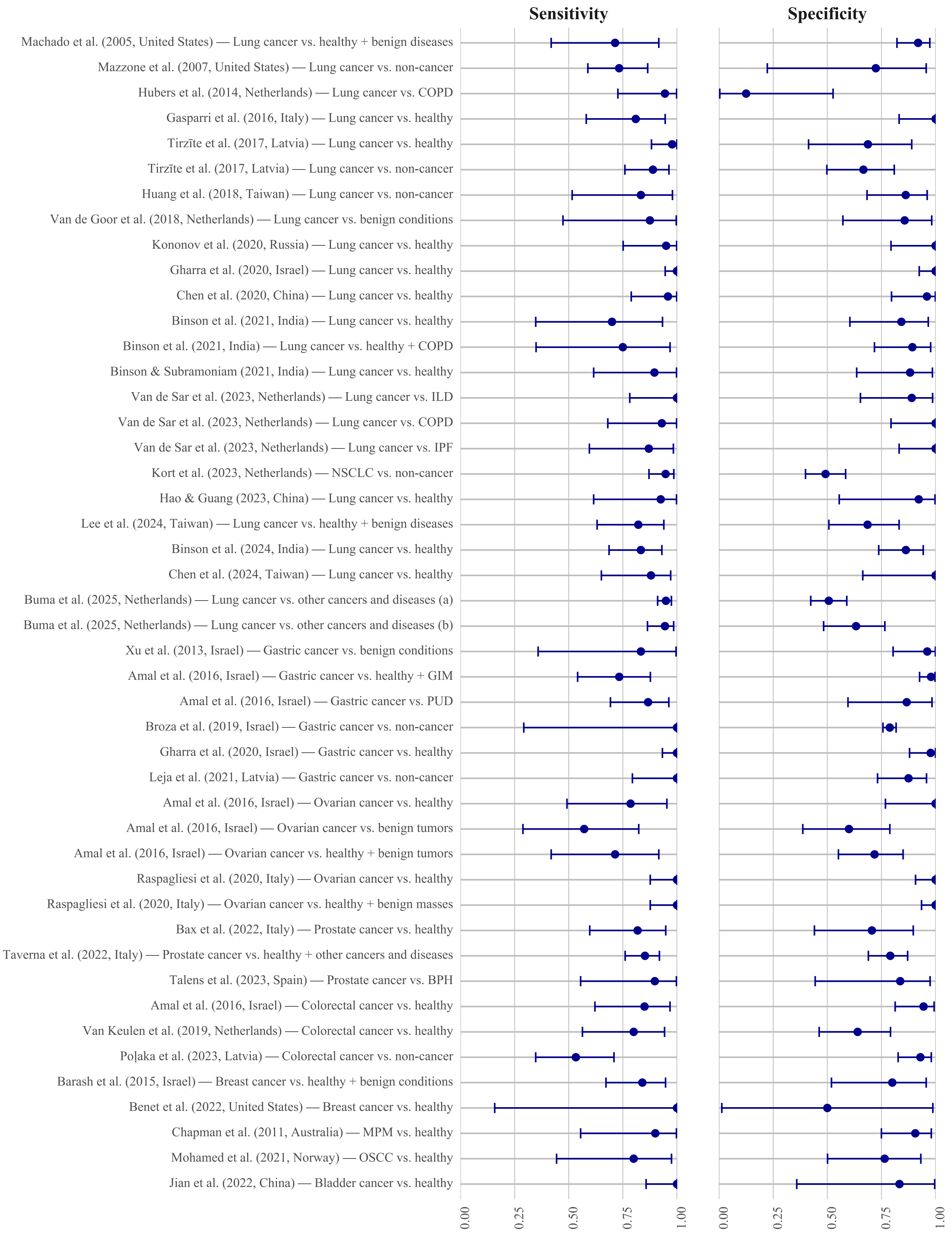

Supplementary Figure S7. Per-Study Sensitivity and Specificity
Bands represent corresponding 95% confidence intervals.

References

1. Portable Electronic Nose | AIRSENSE Analytics. Accessed May 28, 2025. https://airsense.com/en/products/portable-electronic-nose

2. Lung cancer detection with aeoNose. The eNose Company. Accessed May 26, 2025. https://www.enose-company.com/en/disease/lung-cancer/

3. Cyranose 320 Electronic Nose - Smart Smell Detection Sensors. Accessed May 26, 2025. https://www.sensigent.com/cyranose-320.html

4. SpiroNose | Breathomix. Accessed May 28, 2025. https://www.breathomix.com/spironose-2/

5. Di Natale C, Macagnano A, Martinelli E, et al. Lung cancer identification by the analysis of breath by means of an array of non-selective gas sensors. *Biosens Bioelectron*. 2003;18(10):1209-1218. doi:10.1016/S0956-5663(03)00086-1

6. Blatt R, Bonarini A, Calabro E, Torre MD, Matteucci M, Pastorino U. Lung Cancer Identification by an Electronic Nose based on an Array of MOS Sensors. In: *2007 International Joint Conference on Neural Networks*. ; 2007:1423-1428. doi:10.1109/IJCNN.2007.4371167

7. D'Amico A, Pennazza G, Santonico M, et al. An investigation on electronic nose diagnosis of lung cancer. *Lung Cancer*. 2010;68(2):170-176. doi:10.1016/j.lungcan.2009.11.003

8. Hakim M, Billan S, Tisch U, et al. Diagnosis of head-and-neck cancer from exhaled breath. *Br J Cancer*. 2011;104(10):1649-1655. doi:10.1038/bjc.2011.128

9. Wang D, Yu K, Wang Y, et al. A HYBRID ELECTRONIC NOSES' SYSTEM BASED ON MOS-SAW DETECTION UNITS INTENDED FOR LUNG CANCER DIAGNOSIS. *J Innov Opt Health Sci*. 2012;05(01):1150006. doi:10.1142/S1793545811500064

10. Broza YY, Kremer R, Tisch U, et al. A nanomaterial-based breath test for short-term follow-up after lung tumor resection. *Nanomedicine Nanotechnol Biol Med*. 2013;9(1):15-21. doi:10.1016/j.nano.2012.07.009

11. Mazzone PJ, Wang XF, Xu Y, et al. Exhaled Breath Analysis with a Colorimetric Sensor Array for the Identification and Characterization of Lung Cancer. *J Thorac Oncol*. 2012;7(1):137-142. doi:10.1097/JTO.0b013e318233d80f

12. Santonico M, Lucantoni G, Pennazza G, et al. In situ detection of lung cancer volatile fingerprints using bronchoscopic air-sampling. *Lung Cancer*. 2012;77(1):46-50. doi:10.1016/j.lungcan.2011.12.010

13. Peled N, Hakim M, Bunn PA, et al. Non-invasive Breath Analysis of Pulmonary Nodules. *J Thorac Oncol*. 2012;7(10):1528-1533. doi:10.1097/JTO.0b013e3182637d5f

14. Bikov A, Hernadi M, Korosi BZ, et al. Expiratory flow rate, breath hold and anatomic dead space influence electronic nose ability to detect lung cancer. *BMC Pulm Med*. 2014;14(1):202. doi:10.1186/1471-2466-14-202

15. Mazzone PJ, Wang XF, Lim S, et al. Accuracy of volatile urine biomarkers for the detection and characterization of lung cancer. *BMC Cancer*. 2015;15(1):1001. doi:10.1186/s12885-015-1996-0

16. McWilliams A, Beigi P, Srinidhi A, Lam S, MacAulay CE. Sex and Smoking Status Effects on the Early Detection of Early Lung Cancer in High-Risk Smokers Using an Electronic Nose. *IEEE Trans Biomed Eng*. 2015;62(8):2044-2054. doi:10.1109/TBME.2015.2409092

17. Rocco R, Incalzi RA, Pennazza G, et al. BIONOTE e-nose technology may reduce false positives in lung cancer screening programmes. *Eur J Cardiothorac Surg*. 2016;49(4):1112-1117. doi:10.1093/ejcts/ezv328

18. Tan JL, Yong ZX, Liam CK. Using a chemiresistor-based alkane sensor to distinguish exhaled breaths of lung cancer patients from subjects with no lung cancer. *J Thorac Dis*. 2016;8(10):2772-2783. doi:10.21037/jtd.2016.10.30

19. Li W, Liu H, Xie D, He Z, Pi X. Lung Cancer Screening Based on Type-different Sensor Arrays. *Sci Rep*. 2017;7(1):1969. doi:10.1038/s41598-017-02154-9

20. Shlomi D, Abud M, Liran O, et al. Detection of Lung Cancer and EGFR Mutation by Electronic Nose System. *J Thorac Oncol*. 2017;12(10):1544-1551. doi:10.1016/j.jtho.2017.06.073

21. Chang JE, Lee DS, Ban SW, et al. Analysis of volatile organic compounds in exhaled breath for lung cancer diagnosis using a sensor system. *Sens Actuators B Chem*. 2018;255:800-807. doi:10.1016/j.snb.2017.08.057

22. Kort S, Tiggeloven MM, Brusse-Keizer M, et al. Multi-centre prospective study on diagnosing subtypes of lung cancer by exhaled-breath analysis. *Lung Cancer*. 2018;125:223-229. doi:10.1016/j.lungcan.2018.09.022

23. Tirzïte M, Bukovskis M, Strazda G, Jurka N, Taivans I. Detection of lung cancer with electronic nose and logistic regression analysis. *J Breath Res*. 2018;13(1):016006. doi:10.1088/1752-7163/aae1b8

24. Lu B, Fu L, Nie B, Peng Z, Liu H. A Novel Framework with High Diagnostic Sensitivity for Lung Cancer Detection by Electronic Nose. *Sensors*. 2019;19(23):5333. doi:10.3390/s19235333

25. Marzorati D, Mainardi L, Sedda G, Gasparri R, Spaggiari L, Cerveri P. A Metal Oxide Gas Sensors Array for Lung Cancer Diagnosis Through Exhaled Breath Analysis. In: *2019 41st Annual International Conference of the IEEE Engineering in Medicine and Biology Society (EMBC)*. IEEE; 2019:1584-1587. doi:10.1109/EMBC.2019.8856750

26. Mohamed EI, Mohamed MA, Abdel-Mageed SM, Abdel-Mohdy TS, Badawi MI, Darwish SH. Volatile organic compounds of biofluids for detecting lung cancer by an electronic nose based on artificial neural network. *J Appl Biomed*. 2019;17(1):67-67. doi:10.32725/jab.2018.006

27. Fielding D, Hartel G, Pass D, et al. Volatile organic compound breath testing detects in-situ squamous cell carcinoma of bronchial and laryngeal regions and shows distinct profiles of each tumour. *J Breath Res*. 2020;14(4):046013. doi:10.1088/1752-7163/abb18a

28. Kort S, Brusse-Keizer M, Gerritsen JW, et al. Improving lung cancer diagnosis by combining exhaled-breath data and clinical parameters. *ERJ Open Res*. 2020;6(1):00221-02019. doi:10.1183/23120541.00221-2019

29. Krauss E, Haberer J, Barreto G, Degen M, Seeger W, Guenther A. Recognition of breathprints of lung cancer and chronic obstructive pulmonary disease using the Aeonose® electronic nose. *J Breath Res*. 2020;14(4):046004. doi:10.1088/1752-7163/ab8c50

30. Rodríguez-Aguilar M, Díaz De León-Martínez L, Gorocica-Rosete P, et al. Application of chemoresistive gas sensors and chemometric analysis to differentiate the fingerprints of global volatile organic compounds from diseases. Preliminary results of COPD, lung cancer and breast cancer. *Clin Chim Acta*. 2021;518:83-92. doi:10.1016/j.cca.2021.03.016

31. Binson VA, Subramoniam M, Sunny Y, Mathew L. Prediction of Pulmonary Diseases With Electronic Nose Using SVM and XGBoost. *IEEE Sens J*. 2021;21(18):20886-20895. doi:10.1109/JSEN.2021.3100390

32. Binson VA, Subramoniam M, Mathew L. Noninvasive detection of COPD and Lung Cancer through breath analysis using MOS Sensor array based e-nose. *Expert Rev Mol Diagn*. 2021;21(11):1223-1233. doi:10.1080/14737159.2021.1971079

33. Binson VA, Subramoniam M, Mathew L. Discrimination of COPD and lung cancer from controls through breath analysis using a self-developed e-nose. *J Breath Res*. 2021;15(4):046003. doi:10.1088/1752-7163/ac1326

34. Chen K, Liu L, Nie B, et al. Recognizing lung cancer and stages using a self-developed electronic nose system. *Comput Biol Med*. 2021;131:104294. doi:10.1016/j.compbiomed.2021.104294

35. Liu L, Li W, He Z, et al. Detection of lung cancer with electronic nose using a novel ensemble learning framework. *J Breath Res*. 2021;15(2):026014. doi:10.1088/1752-7163/abe5c9

36. Gasparri R, Capuano R, Guaglio A, et al. Volatolomic urinary profile analysis for diagnosis of the early stage of lung cancer. *J Breath Res*. 2022;16(4):046008. doi:10.1088/1752-7163/ac88ec

37. Gruber M, Tisch U, Jeries R, et al. Analysis of exhaled breath for diagnosing head and neck squamous cell carcinoma: a feasibility study. *Br J Cancer*. 2014;111(4):790-798.

doi:10.1038/bjc.2014.361

38. Leunis N, Boumans M, Kremer B, et al. Application of an electronic nose in the diagnosis of head and neck cancer. *The Laryngoscope*. 2014;124(6):1377-1381. doi:10.1002/lary.24463

39. Van De Goor RMGE, Hardy JCA, Van Hooren MRA, Kremer B, Kross KW. Detecting recurrent head and neck cancer using electronic nose technology: A feasibility study. *Head Neck*. 2019;41(9):2983-2990. doi:10.1002/hed.25787

40. Van De Goor RMGE, Van Hooren MRA, Henatsch D, Kremer B, Kross KW. Detecting head and neck squamous carcinoma using a portable handheld electronic nose. *Head Neck*. 2020;42(9):2555-2559. doi:10.1002/hed.26293

41. Anzivino R, Sciancalepore PI, Dragonieri S, et al. The Role of a Polymer-Based E-Nose in the Detection of Head and Neck Cancer from Exhaled Breath. *Sensors*. 2022;22(17):6485. doi:10.3390/s22176485

42. Weber CM, Cauchi M, Patel M, et al. Evaluation of a gas sensor array and pattern recognition for the identification of bladder cancer from urine headspace. *The Analyst*. 2011;136(2):359-364. doi:10.1039/C0AN00382D

43. Heers H. Non-invasive Detection of Bladder Tumors Through Volatile Organic Compounds: A Pilot Study with an Electronic Nose. *Anticancer Res*. 2018;38(2). doi:10.21873/anticanres.12291

44. Matsumoto K, Murakami Y, Shimizu Y, Hirayama T, Ishikawa W, Iwamura M. Electronic nose to distinguish bladder cancer by urinary odour feature: A pilot study. *Cancer Biomark*. 2020;28(1):33-39. doi:10.3233/CBM-190466

45. Zhu S, Huang Z, Nabi G. Fluorometric optical sensor arrays for the detection of urinary bladder cancer specific volatile organic compounds in the urine of patients with frank hematuria: a prospective case-control study. *Biomed Opt Express*. 2020;11(2):1175. doi:10.1364/BOE.380629

46. Bassi P, Di Gianfrancesco L, Salmaso L, et al. Improved Non-Invasive Diagnosis of Bladder Cancer with an Electronic Nose: A Large Pilot Study. *J Clin Med*. 2021;10(21):4984. doi:10.3390/jcm10214984

47. Shuster G, Gallimidi Z, Reiss AH, et al. Classification of breast cancer precursors through exhaled breath. *Breast Cancer Res Treat*. 2011;126(3):791-796. doi:10.1007/s10549-010-1317-x

48. Barash O, Zhang W, Halpern JM, et al. Differentiation between genetic mutations of breast cancer by breath volatolomics. *Oncotarget*. 2015;6(42):44864-44876. doi:10.18632/oncotarget.6269

49. Herman-Saffar O, Boger Z, Libson S, Lieberman D, Gonen R, Zeiri Y. Early

non-invasive detection of breast cancer using exhaled breath and urine analysis. *Comput Biol Med*. 2018;96:227-232. doi:10.1016/j.compbiomed.2018.04.002

50. Díaz De León-Martínez L, Rodríguez-Aguilar M, Gorocica-Rosete P, et al. Identification of profiles of volatile organic compounds in exhaled breath by means of an electronic nose as a proposal for a screening method for breast cancer: a case-control study. *J Breath Res*. 2020;14(4):046009. doi:10.1088/1752-7163/aba83f

51. Asimakopoulos AD, Del Fabbro D, Miano R, et al. Prostate cancer diagnosis through electronic nose in the urine headspace setting: a pilot study. *Prostate Cancer Prostatic Dis*. 2014;17(2):206-211. doi:10.1038/pcan.2014.11

52. Waltman CG, Marcelissen TAT, Van Roermund JGH. Exhaled-breath Testing for Prostate Cancer Based on Volatile Organic Compound Profiling Using an Electronic Nose Device (Aeonose™): A Preliminary Report. *Eur Urol Focus*. 2020;6(6):1220-1225. doi:10.1016/j.euf.2018.11.006

53. Bax C, Bernasconi R, Massironi F, et al. Inkjet Printed ZnO Sensors for Early Prostate Cancer Detection by Means of Urine Odor Analysis. *J Electrochem Soc*. 2021;168(4):047513. doi:10.1149/1945-7111/abf7e7

54. Capelli L, Bax C, Grizzi F, Taverna G. Optimization of training and measurement protocol for eNose analysis of urine headspace aimed at prostate cancer diagnosis. *Sci Rep*. 2021;11(1):20898. doi:10.1038/s41598-021-00033-y

55. Yang HY, Wang YC, Peng HY, Huang CH. Breath biopsy of breast cancer using sensor array signals and machine learning analysis. *Sci Rep*. 2021;11(1):103. doi:10.1038/s41598-020-80570-0

56. Filianoti A, Costantini M, Bove AM, et al. Volatilome Analysis in Prostate Cancer by Electronic Nose: A Pilot Monocentric Study. *Cancers*. 2022;14(12):2927. doi:10.3390/cancers14122927

57. Heers H, Chwilka O, Huber J, et al. VOC‐based detection of prostate cancer using an electronic nose and ion mobility spectrometry: A novel urine‐based approach. *The Prostate*. 2024;84(8):756-762. doi:10.1002/pros.24692

58. De Meij TG, Larbi IB, Van Der Schee MP, et al. Electronic nose can discriminate colorectal carcinoma and advanced adenomas by fecal volatile biomarker analysis: proof of principle study. *Int J Cancer*. 2014;134(5):1132-1138. doi:10.1002/ijc.28446

59. Westenbrink E, Arasaradnam RP, O'Connell N, et al. Development and application of a new electronic nose instrument for the detection of colorectal cancer. *Biosens Bioelectron*. 2015;67:733-738. doi:10.1016/j.bios.2014.10.044

60. Altomare DF, Porcelli F, Picciariello A, et al. The use of the PEN3 e-nose in the screening of colorectal cancer and polyps. *Tech Coloproctology*. 2016;20(6):405-409. doi:10.1007/s10151-016-1457-z

61. Amal H, Leja M, Funka K, et al. Breath testing as potential colorectal cancer screening tool: Breath test for the detection of colorectal cancer. *Int J Cancer*. 2016;138(1):229-236. doi:10.1002/ijc.29701

62. Tyagi H, Daulton E, Bannaga AS, Arasaradnam RP, Covington JA. Non-Invasive Detection and Staging of Colorectal Cancer Using a Portable Electronic Nose. *Sensors*. 2021;21(16):5440. doi:10.3390/s21165440

63. Daniel DaP, Thangavel K. Breathomics for gastric cancer classification using back-propagation neural network. *J Med Signals Sens*. 2016;6(3):172. doi:10.4103/2228-7477.186879

64. Schuermans VNE, Li Z, Jongen ACHM, et al. Pilot Study: Detection of Gastric Cancer From Exhaled Air Analyzed With an Electronic Nose in Chinese Patients. *Surg Innov*. 2018;25(5):429-434. doi:10.1177/1553350618781267

65. Polaka I, Bhandari MP, Mezmale L, et al. Modular Point-of-Care Breath Analyzer and Shape Taxonomy-Based Machine Learning for Gastric Cancer Detection. *Diagnostics*. 2022;12(2):491. doi:10.3390/diagnostics12020491

66. Dragonieri S, Van Der Schee MP, Massaro T, et al. An electronic nose distinguishes exhaled breath of patients with Malignant Pleural Mesothelioma from controls. *Lung Cancer*. 2012;75(3):326-331. doi:10.1016/j.lungcan.2011.08.009

67. Bordbar MM, Barzegar H, Tashkhourian J, Bordbar M, Hemmateenejad B. A non-invasive tool for early detection of acute leukemia in children using a paper-based optoelectronic nose based on an array of metallic nanoparticles. *Anal Chim Acta*. 2021;1141:28-35. doi:10.1016/j.aca.2020.10.029

68. Baudrexler T, Boeselt T, Li L, et al. Volatile Phases Derived from Serum, DC, or MLC Culture Supernatants to Deduce a VOC-Based Diagnostic Profiling Strategy for Leukemic Diseases. *Biomolecules*. 2023;13(6):989. doi:10.3390/biom13060989

69. Evenhuis RE, Acem I, Van Praag VM, Van Der Wal RJ, Bus MP, Van De Sande MA. Diagnosis of chondrosarcoma in a noninvasive way using volatile organic compounds in exhaled breath: a pilot study. *Future Oncol*. 2024;20(22):1545-1552. doi:10.1080/14796694.2024.2355080

70. Díaz De León-Martínez L, Flores-Ramírez R, López-Mendoza CM, et al. Identification of volatile organic compounds in the urine of patients with cervical cancer. Test concept for timely screening. *Clin Chim Acta*. 2021;522:132-140. doi:10.1016/j.cca.2021.08.014

71. Murdocca M, Torino F, Pucci S, et al. Urine LOX-1 and Volatilome as Promising Tools towards the Early Detection of Renal Cancer. *Cancers*. 2021;13(16):4213. doi:10.3390/cancers13164213

72. Costantini M, Filianoti A, Anceschi U, et al. Human Urinary Volatilome Analysis in Renal Cancer by Electronic Nose. *Biosensors*. 2023;13(4):427. doi:10.3390/bios13040427

73. Scheepers MHMC, Al-Difaie ZJJ, Wintjens AGWE, et al. Detection of differentiated thyroid carcinoma in exhaled breath with an electronic nose. *J Breath Res*. 2022;16(3):036008. doi:10.1088/1752-7163/ac77a9

74. Angioli R, Santonico M, Pennazza G, et al. Use of Sensor Array Analysis to Detect Ovarian Cancer through Breath, Urine, and Blood: A Case-Control Study. *Diagnostics*. 2024;14(5):561. doi:10.3390/diagnostics14050561

75. Machado RF, Laskowski D, Deffenderfer O, et al. Detection of Lung Cancer by Sensor Array Analyses of Exhaled Breath. *Am J Respir Crit Care Med*. 2005;171(11):1286-1291. doi:10.1164/rccm.200409-1184OC

76. Mazzone PJ, Hammel J, Dweik R, et al. Diagnosis of lung cancer by the analysis of exhaled breath with a colorimetric sensor array. *Thorax*. 2007;62(7):565-568. doi:10.1136/thx.2006.072892

77. Hubers AJ, Brinkman P, Boksem RJ, et al. Combined sputum hypermethylation and eNose analysis for lung cancer diagnosis. *J Clin Pathol*. 2014;67(8):707-711. doi:10.1136/jclinpath-2014-202414

78. Gasparri R, Santonico M, Valentini C, et al. Volatile signature for the early diagnosis of lung cancer. *J Breath Res*. 2016;10(1):016007. doi:10.1088/1752-7155/10/1/016007

79. Tirzīte M, Bukovskis M, Strazda G, Jurka N, Taivans I. Detection of lung cancer in exhaled breath with an electronic nose using support vector machine analysis. *J Breath Res*. 2017;11(3):036009. doi:10.1088/1752-7163/aa7799

80. Huang CH, Zeng C, Wang YC, et al. A Study of Diagnostic Accuracy Using a Chemical Sensor Array and a Machine Learning Technique to Detect Lung Cancer. *Sensors*. 2018;18(9):2845. doi:10.3390/s18092845

81. Van De Goor R, Van Hooren M, Dingemans AM, Kremer B, Kross K. Training and Validating a Portable Electronic Nose for Lung Cancer Screening. *J Thorac Oncol*. 2018;13(5):676-681. doi:10.1016/j.jtho.2018.01.024

82. Kononov A, Korotetsky B, Jahatspanian I, et al. Online breath analysis using metal oxide semiconductor sensors (electronic nose) for diagnosis of lung cancer. *J Breath Res*. 2019;14(1):016004. doi:10.1088/1752-7163/ab433d

83. Gharra A, Broza YY, Yu G, et al. Exhaled breath diagnostics of lung and gastric cancers in China using nanosensors. *Cancer Commun*. 2020;40(6):273-278. doi:10.1002/cac2.12030

84. Chen Q, Chen Z, Liu D, He Z, Wu J. Constructing an E-Nose Using Metal-Ion-Induced Assembly of Graphene Oxide for Diagnosis of Lung Cancer via Exhaled Breath. *ACS Appl Mater Interfaces*. 2020;12(15):17713-17724. doi:10.1021/acsami.0c00720

85. Binson VA, Subramoniam M, Mathew L. Detection of COPD and Lung Cancer with electronic nose using ensemble learning methods. *Clin Chim Acta*. 2021;523:231-238.

doi:10.1016/j.cca.2021.10.005

86. Binson VA, Subramoniam M. Design and development of an e-nose system for the diagnosis of pulmonary diseases. *Acta Bioeng Biomech*. 2021;23(1). doi:10.37190/ABB-01737-2020-03

87. Van Der Sar IG, Wijsenbeek MS, Braunstahl GJ, et al. Differentiating interstitial lung diseases from other respiratory diseases using electronic nose technology. *Respir Res*. 2023;24(1):271. doi:10.1186/s12931-023-02575-3

88. Kort S, Brusse-Keizer M, Schouwink H, et al. Diagnosing Non-Small Cell Lung Cancer by Exhaled Breath Profiling Using an Electronic Nose. *Chest*. 2023;163(3):697-706. doi:10.1016/j.chest.2022.09.042

89. Hao L, Huang G. An improved AdaBoost algorithm for identification of lung cancer based on electronic nose. *Heliyon*. 2023;9(3):e13633. doi:10.1016/j.heliyon.2023.e13633

90. Lee MR, Kao MH, Hsieh YC, et al. Cross-site validation of lung cancer diagnosis by electronic nose with deep learning: a multicenter prospective study. *Respir Res*. 2024;25(1):203. doi:10.1186/s12931-024-02840-z

91. Binson VA, Mathew P, Thomas S, Mathew L. Detection of lung cancer and stages via breath analysis using a self-made electronic nose device. *Expert Rev Mol Diagn*. 2024;24(4):341-353. doi:10.1080/14737159.2024.2316755

92. Chen KC, Kuo SW, Shie RH, Yang HY. Advancing accuracy in breath testing for lung cancer: strategies for improving diagnostic precision in imbalanced data. *Respir Res*. 2024;25(1):32. doi:10.1186/s12931-024-02668-7

93. Buma AIG, Muntinghe-Wagenaar MB, Noort V van der, et al. Lung cancer detection by electronic nose analysis of exhaled breath: a multicentre prospective external validation study. *Ann Oncol*. 2025;0(0). doi:10.1016/j.annonc.2025.03.013

94. Xu Z q, Broza YY, Ionsecu R, et al. A nanomaterial-based breath test for distinguishing gastric cancer from benign gastric conditions. *Br J Cancer*. 2013;108(4):941-950. doi:10.1038/bjc.2013.44

95. Amal H, Leja M, Funka K, et al. Detection of precancerous gastric lesions and gastric cancer through exhaled breath. *Gut*. 2016;65(3):400-407. doi:10.1136/gutjnl-2014-308536

96. Broza YY, Khatib S, Gharra A, et al. Screening for gastric cancer using exhaled breath samples. *Br J Surg*. 2019;106(9):1122-1125. doi:10.1002/bjs.11294

97. Leja M, Kortelainen JM, Polaka I, et al. Sensing gastric cancer via point‐of‐care sensor breath analyzer. *Cancer*. 2021;127(8):1286-1292. doi:10.1002/cncr.33437

98. Amal H, Shi D, Ionescu R, et al. Assessment of ovarian cancer conditions from exhaled breath. *Int J Cancer*. 2015;136(6). doi:10.1002/ijc.29166

99. Raspagliesi F, Bogani G, Benedetti S, Grassi S, Ferla S, Buratti S. Detection of Ovarian Cancer through Exhaled Breath by Electronic Nose: A Prospective Study. *Cancers*. 2020;12(9):2408. doi:10.3390/cancers12092408

100. Bax C, Prudenza S, Gaspari G, Capelli L, Grizzi F, Taverna G. Drift compensation on electronic nose data for non-invasive diagnosis of prostate cancer by urine analysis. *iScience*. 2022;25(1):103622. doi:10.1016/j.isci.2021.103622

101. Taverna G, Grizzi F, Tidu L, et al. Accuracy of a new electronic nose for prostate cancer diagnosis in urine samples. *Int J Urol*. 2022;29(8):890-896. doi:10.1111/iju.14912

102. Talens JB, Pelegri-Sebastia J, Sogorb T, Ruiz JL. Prostate cancer detection using e-nose and AI for high probability assessment. *BMC Med Inform Decis Mak*. 2023;23(1):205. doi:10.1186/s12911-023-02312-2

103. Van Keulen KE, Jansen ME, Schrauwen RWM, Kolkman JJ, Siersema PD. Volatile organic compounds in breath can serve as a non‐invasive diagnostic biomarker for the detection of advanced adenomas and colorectal cancer. *Aliment Pharmacol Ther*. 2020;51(3):334-346. doi:10.1111/apt.15622

104. Poļaka I, Mežmale L, Anarkulova L, et al. The Detection of Colorectal Cancer through Machine Learning-Based Breath Sensor Analysis. *Diagnostics*. 2023;13(21):3355. doi:10.3390/diagnostics13213355

105. Giró Benet J, Seo M, Khine M, Gumà Padró J, Pardo Martnez A, Kurdahi F. Breast cancer detection by analyzing the volatile organic compound (VOC) signature in human urine. *Sci Rep*. 2022;12(1):14873. doi:10.1038/s41598-022-17795-8

106. Chapman EA, Thomas PS, Stone E, Lewis C, Yates DH. A breath test for malignant mesothelioma using an electronic nose. *Eur Respir J*. 2012;40(2):448-454. doi:10.1183/09031936.00040911

107. Mohamed N, Goor R van de, El-Sheikh M, et al. Feasibility of a Portable Electronic Nose for Detection of Oral Squamous Cell Carcinoma in Sudan. *Healthcare*. 2021;9(5):534. doi:10.3390/healthcare9050534

108. Jian Y, Zhang N, Liu T, et al. Artificially Intelligent Olfaction for Fast and Noninvasive Diagnosis of Bladder Cancer from Urine. *ACS Sens*. 2022;7(6):1720-1731. doi:10.1021/acssensors.2c00467
